# The Clumped Isotope Signatures of Multiple Methanogenic Metabolisms

**DOI:** 10.1021/acs.est.5c03255

**Published:** 2025-07-03

**Authors:** Jiawen Li, Jeanine L. Ash, Alec Cobban, Briana C. Kubik, Gabriella Rizzo, Mia Thompson, Laetitia Guibourdenche, Stefanie Berger, Kaycee Morra, Ying Lin, Elliott P. Mueller, Andrew L. Masterson, Rebekah Stein, Marilyn Fogel, Mark A. Torres, Xiahong Feng, James F. Holden, Anna Martini, Cornelia U. Welte, Mike S. M. Jetten, Edward D. Young, William D. Leavitt

**Affiliations:** † Department of Earth Sciences, 3728 Dartmouth College, Hanover, New Hampshire 03755, United States; ‡ Department of Earth, Environmental and Planetary Sciences, 3990 Rice University, Houston, Texas 77005, United States; § Department of Microbiology, 14707 University of Massachusetts Amherst, Amherst, Massachusetts 01003, United States; ∥ Department of Earth, Planetary, and Space Sciences, 158087 University of California, Los Angeles, Los Angeles, California 90095, United States; ⊥ Department of Microbiology, RIBES, Radboud University, Nijmegen 6525 XZ, the Netherlands; # Department of Earth and Planetary Sciences, EDGE Institute, 6029 University of California, Riverside, Riverside, California 92521, United States; ∇ Division of Geological and Planetary Sciences, California Institute of Technology, Pasadena, California 91125, United States; ○ Department of Earth and Planetary Sciences, 3270 Northwestern University, Evanston, Illinois 60208, United States; ◆ Cooperative Programs for the Advancement of Earth System Science, 1879 University Corporation for Atmospheric Research, Boulder, Colorado 80307, United States; ¶ Department of Geology, 1180 Amherst College, Amherst, Massachusetts 01002, United States; k Department of Geology & Geophysics, University of Utah, Salt Lake City, Utah 84112, United States; l Department of Chemistry, Dartmouth College, Hanover, New Hampshire 03755, United States

**Keywords:** methane, methanogenesis pathways, clumped isotopologues, combinatorial effect

## Abstract

Methane is a potent greenhouse gas, an important energy source, and an important part of the global carbon cycle. The relative abundances of doubly substituted (“clumped”) methane isotopologues (^13^CH_3_D and ^12^CH_2_D_2_) offer important information on the sources and sinks of methane. However, the clumped isotope signatures of microbially produced methane from different methanogenic pathways lack a systematic investigation. In this study, we provide a data set encompassing isotopic signatures of hydrogenotrophic, methylotrophic, acetoclastic, and methoxydotrophic methanogenesis. We find that a statistical “combinatorial effect” generates significant differences in ^12^CH_2_D_2_ compositions between hydrogenotrophic methanogenesis and the other pathways, while variations in the fractionation factors of clumped isotopologues result in differences in ^13^CH_3_D compositions between the methylotrophic, acetoclastic, and methoxydotrophic pathways. The energy yield of methanogenesis and the energy conservation approaches implemented by different microbial strains may also influence the isotope values of methane. Further analysis suggests that previously observed isotopic signatures of methane in freshwater environments are potentially due to mixing between hydrogenotrophic and other methanogenesis pathways. This study provides new experimental constraints on the isotope signatures of different microbial methanogenic pathways and evidence of the mechanisms responsible for the observed differences. This enables a better understanding of the sources and sinks of methane in the environment.

## Graphical abstract

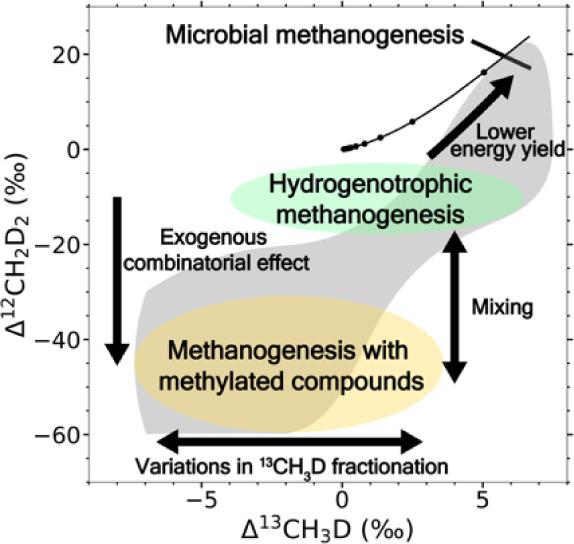

## Introduction

Methane is a critical energy source, a greenhouse gas, and a key component of the global carbon cycle. Tracking the sources and sinks of methane is crucial for reconstructing the methane budget and carbon cycle on Earth.^1^ Methane is produced by both biological and nonbiological processes.^1,2^ Biogenic methane produced by microbes encompasses a spectrum of biochemical pathways that utilize a variety of substrates. The three major microbial methanogenesis pathways are hydrogenotrophic (H_2_/CO_2_), methylotrophic (methanol, methylamine, or methyl sulfide, among others), and acetoclastic (acetate) methanogenesis.^3^ More rare yet important biogenic pathways include methoxydotrophy^4^ and alkylotrophic methanogenesis.^5^ These pathways are dominant in different environments and can occur simultaneously in nature.^3,6–8^ A challenge for quantifying sources and sinks of methane is accurately tracing of the contributions of various metabolic pathways to biogenic methane fluxes in natural environments.

The carbon and hydrogen isotopic ratios of methane molecules offer useful information on the sources of methane. The bulk isotope compositions of methane (^13^C/^12^C and D/H) have been traditionally used to identify its origins.^9,10^ However, bulk isotope values are influenced by both the isotope compositions of the source materials (e.g., methanol/acetate/CO_2_, H_2_O) that exchange C and H atoms with methane molecules, as well as the isotopic discrimination of carbon and hydrogen during each formation pathway.^11^ More recently, the relative abundance of the doubly substituted (referred to as “clumped” hereafter) isotopologues of methane (^13^CH_3_D and ^12^CH_2_D_2_) have been applied to identify methane from different sources.^12–14^ Unlike bulk isotopes, the clumped isotopologue compositions of methane are not influenced by the isotope values of the source materials, and only depend on the geochemical processes that methane gases undergo.^11^ At thermodynamic equilibrium, the clumped isotopologue compositions of methane are determined by the equilibrium temperature. However, microbial methane produced in laboratory experiments often shows nonequilibrium clumped isotope signatures,^14–17^ whereas microbial methane from some natural environments exhibits clumped isotope values closer to thermodynamic equilibrium at ambient temperature.^12,13,15,18,19^ This discrepancy is proposed to result from the low energy yield during microbial methanogenesis in some natural settings (e.g., marine sediments, mines) under substrate limitation and high pressure.^20^ In contrast, high energy yield conditions are common in lab experiments and presumably, in some terrestrial settings (e.g., wetlands, landfills).^12,13,21,22^

Despite several pure-culture methanogenesis experiments in which multiply substituted isotopologues have been measured,^14–17,23^ there is the need for a better understanding of the effects of different metabolic pathways on isotope clumping. This is very important, both for the interpretation of the sources of methane in nature and for setting the endmembers for modeling postmethanogenic alterations (e.g., methane oxidation, mixing, isotopic re-equilibration). Previous studies mainly focus on the fractionation mechanisms of hydrogenotrophic methanogenesis,^21,22,24^ leaving the other two major biogenic pathways—methylotrophic and acetoclastic methanogenesis—under constrained. Moreover, a recent report highlights the importance of the “combinatorial effect” (described in detail in the Results and Discussion and the Supporting Information) on the clumped isotope signatures of methanogenesis using methylated compounds.^17^ In this study, we present a compilation of the isotopic (bulk and clumped) signatures of methanogenic pathways: hydrogenotrophic, methylotrophic, acetoclastic, and methoxydotrophic methanogenesis, with two major goals. First, we investigate if there are differences between the isotopic signals of the microbial methanogenic pathways. Second, we elucidate the mechanisms for the observed isotopic difference between methanogenic pathways. Furthermore, we highlight the significance of our findings in interpreting the sources of methane in natural environments, especially where the energy yield of methanogenesis is high.

## Materials and Methods

### Microbial Cultivation and Methane Production

 strain WWM603 (referred to as hereafter) was acquired from the Metcalf lab at the University of Illinois^25–27^ and grown in HS medium at Dartmouth College (see the Supporting Information). Basal HS medium was amended with either 125 mM methanol, 40 mM acetate, or no exogenous source (only bicarbonate in the basal medium) in a Coy anaerobic chamber. Following standard medium preparation, 10 mL of media containing each energy source were aliquoted into 26 mL Balch tubes, then sealed with blue butyl septa and crimped with aluminum seals, with six replicates for each electron donor condition. The headspace of each Balch tube was sparged for 5 min with 80:20 (v/v) N_2_:CO_2_ and pressurized to 25 psi before autoclaving. Cooled tubes were inoculated with 200 μL of culture in the late logarithmic growth phase (OD_600_ = 0.27) from hydrogenotrophic growth with no deuterium spike. The samples without any energy source were pressurized to 25 psi with 80:20 (v/v) H_2_:CO_2_ after inoculation. was also cultivated in deuterium-spiked (D-spiked) medium water, where the nominated δD values of water were +3000 or +8000 ‰ (V-SMOW). All experiments were inoculated in quintuplicate with a single abiotic control per condition. The samples were then incubated at 35 °C for 92 days (2208 h), after which the tubes were killed with 100 μL of 1 M HCl, and stored inverted (septa down) at 4 °C until isotopic measurements. The abiotic controls were sampled after incubation to confirm the deuterium content of each media water. The final headspace methane amount in the headspace was quantified via gas chromatography (GC), using the method described in the previous literature.^28^

Three strains of methanogens, , *and* (referred to as , and , respectively), were cultured in-batch at Radboud University, using methanol (CH_3_OH), trimethylamine (TMA) and 3,4,5-trimethoxybenzoate (TMB) as the carbon sources. The medium for culturing were prepared with the reagents documented in the Supporting Information. Experiments were performed in quadruplicate 125 mL sterile glass serum bottles closed with red butyl stoppers and aluminum crimp caps. Prior to the actual experiment, 1 mL of culture media was passed into fresh, sterile media up to 4 times to ensure that the final methane measured was produced from the substrate of interest for each experiment. The incubation temperatures were 37, 39, and 65 °C for , and , respectively, and the incubation time of the experiments ranged from 55 to 216 h (Table S2). Methane production over time was monitored via GC. After each experiment, cultures were sacrificed by the injection of 10 mL of 6 M NaOH. Samples were then stored in a cool, dark laboratory cabinet until isotopic measurements.

Monocultures of (), () were grown at 80 °C, and () were grown at 65 °C with varying amounts of H_2_ in the headspace at the University of Massachusetts, Amherst, using the method described in the previous literature.^29,30^ Each 60 mL serum bottle contains 25 mL of DSM 282 growth medium (see the Supporting Information). The headspace was filled with gas under one of the three conditions: 1. flushed and topped off with 80:20 (v/v) H_2_:CO_2_ (referred to as full H_2_); 2. flushed and topped off with 80:20 (v/v) N_2_:CO_2_, and then 30 mL of headspace was removed and replaced with 30 mL of 80:20 (v/v) H_2_:CO_2_ (referred to as 30 mL H_2_); 3. flushed and topped off with 80:20 (v/v) N_2_:CO_2_, and then 10 mL of headspace was removed and replaced with 10 mL of 80:20 (v/v) H_2_:CO_2_ (referred to as 10 mL H_2_). Before incubation, an additional 100 kPa of either H_2_:CO_2_ (for full H_2_ condition) or N_2_:CO_2_ (for 30 and 10 mL H_2_ conditions) was added to each bottle to maintain pressure above ambient. For most conditions, three biological replicates and controls were prepared, except for at 10 mL H_2_, where four biological replicates were made. Experiments with full H_2_ were incubated for 3 to 5 h and the experiments with 30 and 10 mL of H_2_ were incubated for 5 to 8 h and 10 to 15 h, respectively (Table S2). The final methane production was quantified following a similar procedure as described for .

### Isotope Notation

Bulk carbon and hydrogen stable isotope ratios are reported in delta (δ) notation as per mil (‰) differences from the international standards VPDB for C-isotopes and VSMOW for H-isotopes, in eqs 1 and 2, respectively.

1 δC13=103((C13/C12)sample(C13/C12)VPDB−1)

2 $$\delta D=10^{3}\left(\frac{{\left(D/H\right)}_{\mathrm{sample}}}{{\left(D/H\right)}_{\mathrm{VS}\mathrm{MOW}}}-1\right)$$

The two mass-18 multiply substituted isotopologues of methane (^13^CH_3_D and ^12^CH_2_D_2_) are reported in capital delta (Δ) notation in per mil (‰) relative to a stochastic distribution of isotopologues at infinite temperature, as shown by eqs 3 and 4, where the bracketed values are the abundances of the isotopologues.

3 ΔCH3D13=103(([CH3D13]/[CH412])sample([CH3D13]/[CH412])stochastic−1)

4 ΔCH212D2=103(([CH212D2]/[CH412])sample([CH212D2]/[CH412])stochastic−1)

The isotope fractionation factors in the experiments are expressed as α_A‑B_, which denotes the ratio between the heavy to light isotope ratios in two reservoirs A and B:

5 $$\alpha_{\mathrm{A-B}}=\frac{R_{A}}{R_{B}}$$

For methane specifically, *R* denotes the ^13^C/^12^C or D/H ratios. To conveniently compare with earlier studies, we also converted α_A‑B_ into ε_A‑B_:

6 $$\in_{\mathrm{A-B}}=10^{3}\left(\alpha_{A-B}-1\right)$$

In this study, we focused on the carbon isotope fractionation between methane and the carbon sources, as well as hydrogen isotope fractionation between methane and water.

### Methane Isolation and Purification

Methane samples were purified on a vacuum purification system using a GC (SRI 8610C GC-TCD) before being analyzed for methane clumped isotopes. Depending on the concentration of methane, 2–5 mL (for serum bottles) or 15 mL (for Balch tubes) of the headspace gas sample is passively introduced into the vacuum line with a gastight syringe, followed by the purification protocol described in previous studies.^14,17^ The purified methane gas was transferred into a glass finger vial with silica gel at liquid nitrogen temperature, then introduced into the mass spectrometer.

### Isotopic Measurements

The two multiply substituted mass-18 isotopologues of methane (^13^CH_3_D and ^12^CH_2_D_2_) were measured on a Nu Instrument Panorama high-mass-resolution multiple-collector isotope ratio mass spectrometer at the Department of Earth, Planetary, and Space Sciences at the University of California, Los Angeles. The mass spectrometry method follows the application in the previous studies.^14,17,31–36^ Two individual sets of analytical methods were used. In the first set, ^12^CH_3_D/^12^CH_4_ and ^12^CH_2_D_2_/^12^CH_4_ were measured as δD and Δ^12^CH_2_D_2_ for 40 blocks of 20 pairs of sample-reference gas measurement cycles. In the second set, ^13^CH_4_/^12^CH_4_ and ^13^CH_3_D/^12^CH_4_ were measured as δ^13^C and Δ^13^CH_3_D for 20 blocks of 20 pairs of sample-reference gas measurement cycles. Integration time for each sample or reference gas measurement was 30 s. The number of blocks for small-size samples (<100 μmol) were reduced to ensure enough gas for both sets of analytical methods.

Aliquots of Utica gas (a thermogenic gas used as standard at UCLA) were run during and between the analytical sessions from 2016 to 2025. During these sessions, the median of the internal precision (2σ) was 0.014, 0.044, 0.34, and 1.36 ‰ for δ^13^C, δD, Δ^13^CH_3_D and Δ^12^CH_2_D_2_, respectively (*n* = 31). The external precision (2σ) based on repeated purification and isotopic measurements of Utica gas was determined to be 0.377, 0.956, 1.15, and 2.02 ‰ for δ^13^C, δD, Δ^13^CH_3_D and Δ^12^CH_2_D_2_, respectively (*n* = 31).

The measurement procedures of bulk carbon and hydrogen isotope values (δ^13^C and δD) of water and substrates are described in the Supporting Information. Additionally, the clumped isotope values (Δ^12^CHD_2_ and Δ^13^CH_2_D) of the methyl group in the methanol used in the incubations at Dartmouth were measured following the protocol detailed in the Supporting Information.

### Model Design and Implementation

To model the combinatorial effect on the measured clumped isotope signatures, we calculated the abundance of methane isotopologues during methanogenesis using the reaction scheme shown in Table S1. This reaction scheme includes 16 methanogenic reactions between the isotopologues of the methyl group in the substrates and the hydrogen atom from water. The relative production rates of methane isotopologues in Table S1 consist of three parts: abundances of isotopologues of the methyl group and water (bracketed values), carbon and hydrogen fractionation factors (^13^α, ^D^α_p_, and ^D^α_s_), and primary and secondary clumped isotopologue factors (^13CD^γ_p_, ^13CD^γ_s_, ^DD^γ_p_, and ^DD^γ_s_). Clumped isotopologue factors in methanogenesis are introduced to express the deviation from the rule of geometric mean.^13,37^ In general, they reflect the difference between the fractionation factors of the clumped isotopologues (^13CH3D^α and ^12CH2D2^α) and the products of the fractionation factors of the two heavy isotopes in these isotopologues (^13^α^D^α and ^D^α^D^α). Similar to fractionation factors, the “*p*” and “*s*” in the subscripts of γ represent primary and secondary clumped isotopologue effects, respectively. A detailed description of the parameters in the model is in the Supporting Information. The relative production rates of isotopologues in Table S1 are treated as equivalent to the relative abundance of isotopologues in the system, which are used to calculate the isotopic values.

The model for the combinatorial effect is applied to fit the data of D-spiked methylotrophic and acetoclastic experiments at Dartmouth College (Table S2) by tuning the parameters described above (Table S3). The details in obtaining the best-fit values of the parameters are described in the Supporting Information. To evaluate the effect of uncertainties on the modeled results, we conducted a Monte Carlo error propagation (MCEP) with the input parameters and uncertainties. Each parameter in the model is assumed to have a normal distribution with the measured or assigned mean value and 1-σ uncertainty. For each of the two methanogenic pathways, we ran the model using δD_H2O_ as an independent variable, ranging from −150 to +9000 ‰ with a 50 ‰ increment. At each δD_H2O_, we ran 1000 simulations and calculated the mean and standard deviations of the modeled isotopic results.

## Results and Discussion

Our experiments are categorized into nine types based on metabolism and growth conditions (Figure 1, Table S2): H_2_/CO_2_ (35 °C), H_2_/CO_2_ (65–80 °C), CH_3_OH + H_2_, CH_3_OH (35–39 °C), CH_3_OH (65 °C), TMA, TMA + H_2_, TMB, and acetate. The methanogenesis pathways encompass hydrogenotrophic (H_2_/CO_2_), methylotrophic (CH_3_OH, TMA), methoxydotrophic (TMB) and acetoclastic (acetate) methanogenesis. The amount of CH_4_ produced in each culture primarily tracks the amount of substrate provided (Table S2). Due to small differences in the starting growth conditions (e.g., differences in oxygen intrusion during transfer, variations in the number of cells during inoculation, etc.), the net methane production rates vary between biological replicates, resulting in end-point methane production varying between replicates (Table S2). Additionally, the acetoclastic methanogenesis with D-spiked water generates less methane with increasing δD_H2O_, potentially resulting from slower reaction rates with more isotopically heavy water. The clumped isotope signatures we measure for each experiment with natural abundance (i.e., non-D-spiked) water fall within the nominal “microbial methane” field defined in previous studies.^11,14,38,39^ While the bulk isotope signatures exhibit large variations among methanogenic pathways, they are generally within the previously defined range for biogenic methane.^10,40^ The methane δD ranges from −536.88 ± 0.03 to −269.49 ± 0.03‰, and δ^13^C ranges from −108.73 ± 0.006 to −39.13 ± 0.005‰ (Figure 2B, Table S2). The Δ^13^CH_3_D values in this study range from −9.24 ± 0.19 to 3.99 ± 0.15‰ and Δ^12^CH_2_D_2_ values range from −51.85 ± 0.83 to −6.27 ± 0.84‰ (Figure 2A, Table S2). The isotopic compositions of the samples in this study demonstrate pathway and strain dependence, characterized by significant differences in δD and Δ^12^CH_2_D_2_ among different methanogenic pathways (Figure 2, Table S2).

**Figure 1:**
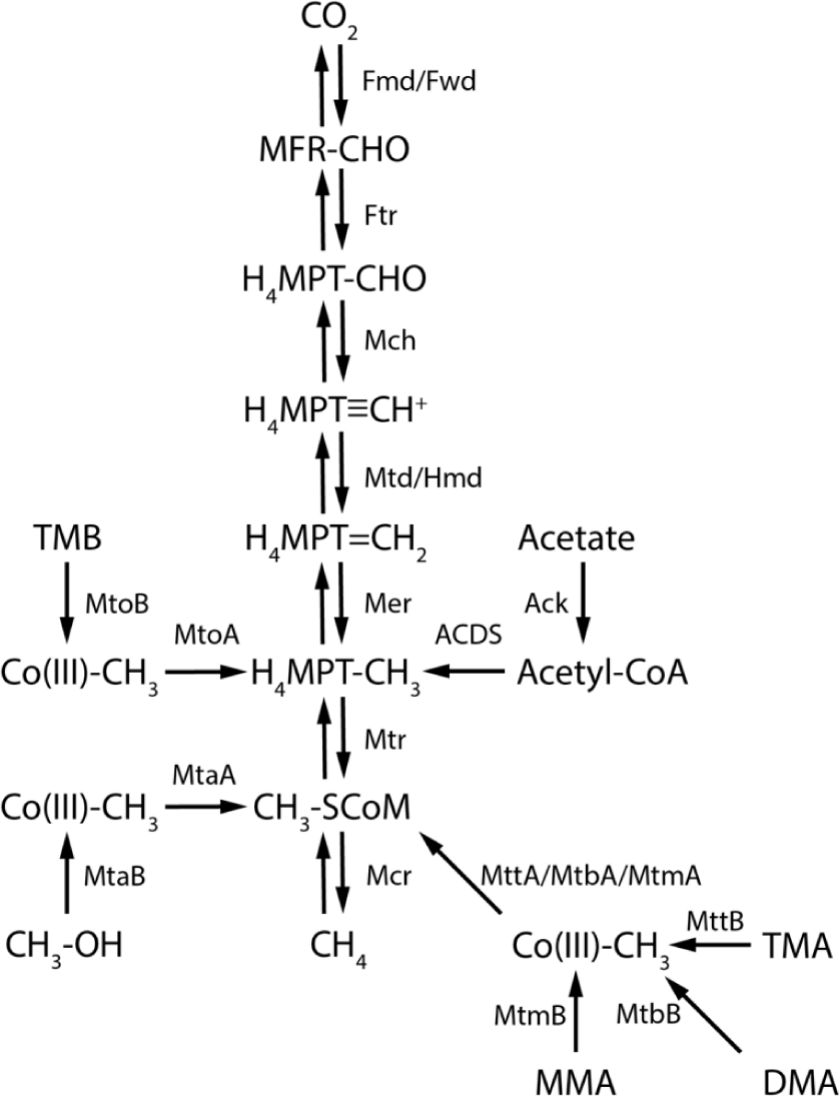
Reaction schemes of the methanogenesis pathways in this study. The reaction scheme is adopted from previous studies,^21,55,69,70^ with the key enzymes and chemical compounds shown on this figure. MFR: methanofuran, H_4_MPT: tetrahydromethanopterin, CoM: coenzyme M, Co­(III): cobalamin binding protein, TMB: 3,4,5-trimethoxybenzoate, TMA: trimethylamine, DMA: dimethylamine, MMA: monomethylamine, Fwd/Fmd: formylmethanofuran dehydrogenase, Ftr: formylmethanofurantetrahydromethanopterin formyl-transferase, Mch: methenyltetrahydromethanopterin cyclohydrolase, Mtd: methylenetetrahydromethanopterin reductase, Hmd: H_2_-forming methylenetetrahydromethanopterin dehydrogenase, Mtr: tetrahydromethanopterin S-methyl-transferase, Mcr: methyl-coenzyme M reductase, Ack: acetate kinase, ACDS: Acetyl-CoA decarbonylase/synthase. Mto A-B, Mta A-B, Mtt A-B, Mtb A-B, Mtm A-B are methyltransferases specific to the methylated compounds. For H_2_-dependent methylotrophic methanogenesis (experiments with CH_3_OH + H_2_ and TMA + H_2_ in this study), the microbes lack the enzymes that catalyze the oxidative reaction branch between CO_2_ and CH_3_–SCoM.^55^

**Figure 2:**
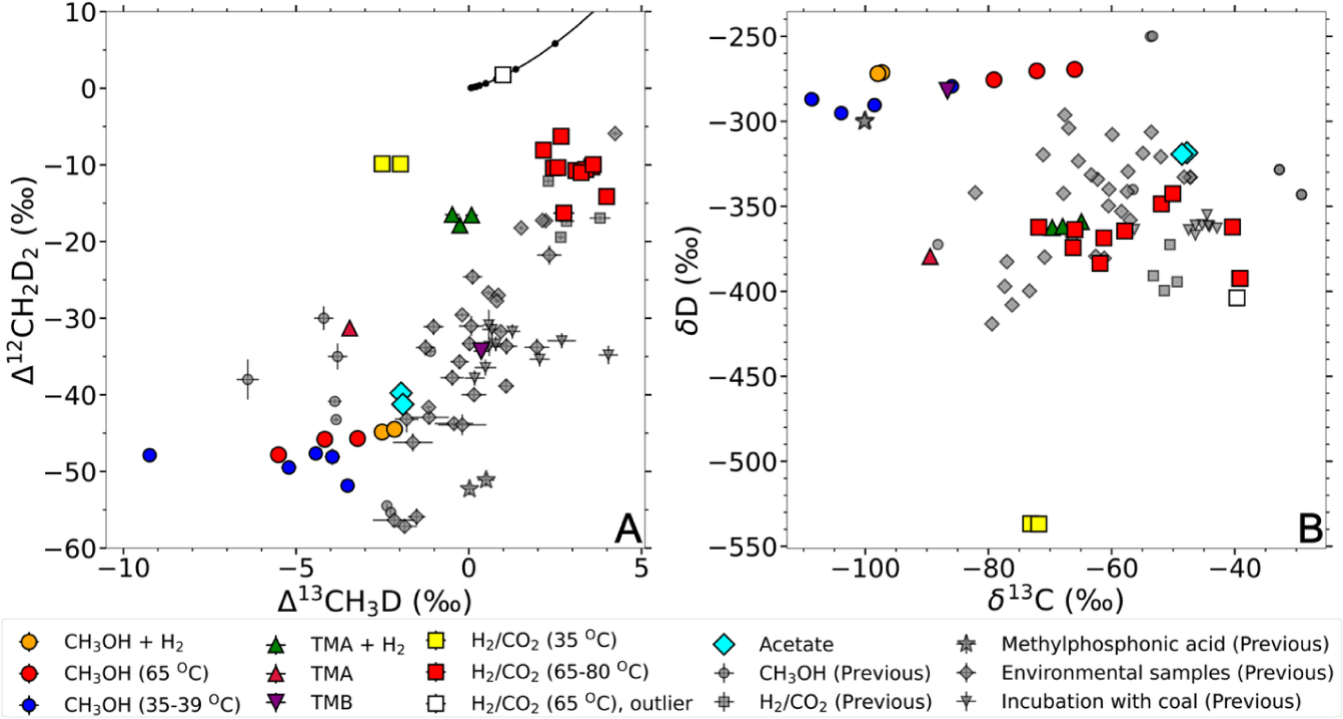
Isotopic data for the non-D-spiked experiments in this study (colored) and previous studies (gray). (A) Clumped isotope signatures; (B) Bulk isotope signatures. The δ^13^C and δD are measured relative to VPDB and VSMOW, respectively. The data used in this figure are shown in Table S1. Previous data from lab incubations, and environmental samples from lakes and wetlands^14–17,39,64–68^ are shown as a reference. The solid black line in panel A is the thermodynamic equilibrium curve for the doubly substituted methane isotopologues. All data included in this figure have both Δ^13^CH_3_D and Δ^12^CH_2_D_2_ values, and show disequilibrium clumped isotope signatures. One biological replicate of hydrogenotrophic methanogenesis by (white square) with full H_2_ is different from the other two replicates under the same growth condition, therefore it is marked as an outlier.

The apparent hydrogen isotope fractionation factors between methane and water (^D^α_CH4‑H2O_) and carbon isotope fractionation factors between methane and source carbon (^13^α_CH4‑Carbon_) are shown in Figure S1 and Table S4. The δD values of waters used in the calculations are listed in Table S2, and the isotopic values of other substrates (CO_2_, CH_3_OH, acetate, TMA, TMB) are listed in Table S5. The fractionation factors are broadly dependent on the methanogenesis pathways. Hydrogenotrophic methanogenesis generates the largest D/H fractionation (^D^α_CH4‑H2O_ between 0.49 and 0.69), followed by methylotrophic methanogenesis with TMA and TMA+H_2_ (^D^α_CH4‑H2O_ between 0.65 and 0.67). Methoxydotrophic, methylotrophic, and acetoclastic methanogenesis with TMB, CH_3_OH, CH_3_OH+H_2_, and acetate yield smaller D/H fractionations (^D^α_CH4‑H2O_ between 0.72 and 0.77). All the hydrogen fractionation factors deviate from the equilibrium values at the incubation temperatures (Figure S1A), suggesting the domination of kinetic isotope effects that are typical in laboratory culture experiments and some freshwater environments. The available ^13^α_CH4‑Carbon_ data show little variation between different methanogenic pathways, ranging from 0.93 to 0.98 (Figure S1B, Table S4).

In the following sections we discuss the patterns of bulk and clumped isotope signatures as a function of microbial methanogenesis pathways, the mechanisms for the observed differences among pathways, and how these may be applied to interpret sources of methane in nature.

### Exogenous Combinatorial Effect Generates Negative Δ^12^CH_2_D_2_ Values

The most distinctive pattern is the difference in Δ^12^CH_2_D_2_ values between hydrogenotrophic and other methanogenic pathways (Figure 2A). This is due to a “combinatorial effect” which originates from the calculation of Δ^12^CH_2_D_2_ in the stochastic reference frame^41,42^ (detailed in the Supporting Information). The combinatorial effect results in negative shifts in Δ^12^CH_2_D_2_ when hydrogen atoms from two or more sources with different δD combine to form methane molecules. The sources of distinct D/H ratios can be intracellular due to fractionation at each enzymatic step^11,24^ or from different external sources of hydrogen (referred to as the “endogenous” and “exogenous” combinatorial effect, respectively). While the effect can alter Δ^12^CH_2_D_2_ values, it does not alter Δ^13^CH_3_D values. The extent of the shift reflects the offset between the δD values of the two (or more) hydrogen pools that contribute to methane formation, with a larger offset producing a more significant negative shift.^41,42^

Hydrogenotrophic methanogens synthesize methane from carbon dioxide and water, using the electrons from molecular hydrogen (H_2_). Compared with the other microbial pathways, hydrogenotrophic methanogens make methane with the most negative δD_CH4_ values, but the least negative Δ^12^CH_2_D_2_ values (Figure 2A). In hydrogenotrophic methanogenesis, all four hydrogen atoms originate from one reservoir, water.^23^ This is supported by the near-zero intercept of the regression line between δD_CH4_ + 1000 and δD_H2O_ + 1000 in Figure S2A, and is consistent with a previous report.^23^ Due to the hydrogen isotope fractionation during each hydrogen addition step or the formation of hydrogen-carrying species (e.g., F_420_H_2_ or HS-CoB), there is a difference between the δD values of hydrocarbon compounds, and the hydrogen atom added. This results in a negative shift in Δ^12^CH_2_D_2_, but the difference is not large enough to produce a significant shift in comparison to other pathways.^14,21,22,24^ Therefore, hydrogenotrophic methanogenesis is characterized by modest Δ^12^CH_2_D_2_ depletions, due to the “endogenous” combinatorial effect.^17^ The dominance of the endogenous combinatorial effect is further confirmed by the D-spiked hydrogenotrophic experiments, where both Δ^13^CH_3_D and Δ^12^CH_2_D_2_ do not change significantly, while δD_CH4_ covaries with δD_H2O_ (Figure 3B-D).

**Figure 3:**
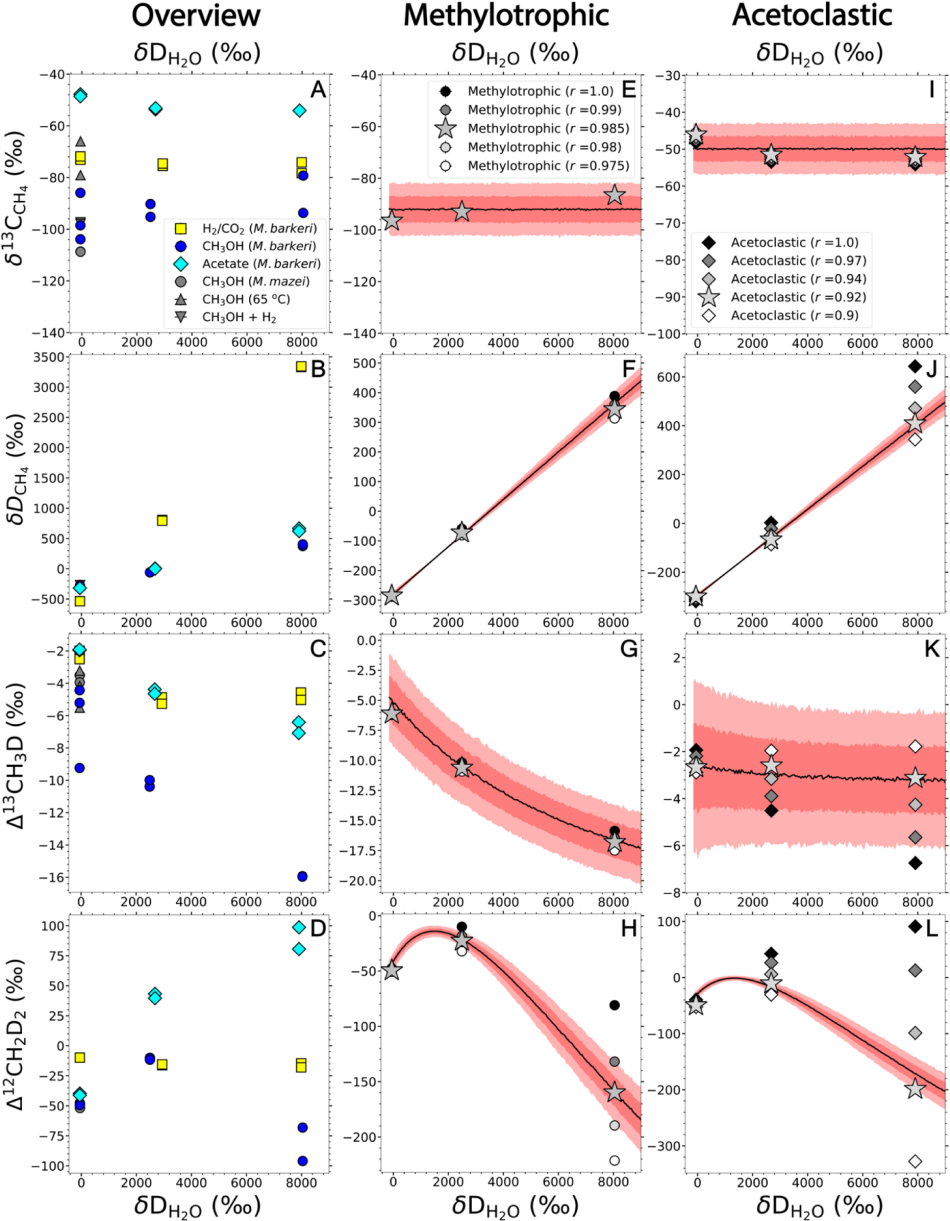
Isotopic results from the D-spiked experiments (panels A–D) and the models for the combinatorial effect in methylotrophic (panels E–H) and acetoclastic (panels I–L) methanogenesis. The δ^13^C and δD values are expressed relative to VPDB and VSMOW. The data from D-spiked experiments at Dartmouth College are shown as colored points. For comparison, the data from non-D-spiked methylotrophic experiments at Radboud are shown as gray points in panels A–D. The measured signals are assumed to be mixtures of methylotrophic or acetoclastic methanogenesis and hydrogenotrophic methanogenesis. In panels E–L, each panel shows the mean original measured isotope signatures in black, and deconvolved isotope signatures of the “pure” methylotrophic or acetoclastic endmembers as stars. The mixing ratio *r* denotes the fraction of the methylotrophic or acetoclastic endmember in the mixture. As references, the isotope values derived from a range of *r* values are shown in each panel. The black lines are the mean values from the model assuming a “pure” combinatorial effect, and the deep and light red areas are the 1-σ and 2-σ uncertainty areas, respectively. The reaction schemes, parameters and description of the model are shown in the Supporting Information.

In contrast, methylotrophic, methoxydotrophic, and acetoclastic methanogenesis yield more negative Δ^12^CH_2_D_2_ and less negative δD_CH4_ values (Figure 2). As demonstrated in the D-spike experiments, this is most likely due to the “exogenous” combinatorial effect,^17^ where some hydrogen atoms on product methane originate from the methyl group in reactants (e.g., methanol, acetate), while the rest source from cellular water. Similar exogenous combinatorial effects have been observed during microbial methane formation from methylphosphonate,^17^ and during thermogenic methane formation by pyrolysis.^38^

For methylotrophic and acetoclastic methanogenesis pathways, three hydrogen atoms in the methane molecule come from the methyl group of methanol or acetate, while the other one hydrogen comes from water (Figure 1). In an ideal scenario, there is no exchange of hydrogen between water and methyl group. This is referred to as a “pure” exogenous combinatorial effect in the following discussion. However, in the actual methanogenesis, a little more than one out of four hydrogen comes from water, due to the reversibility of the dehydrogenation steps from CH_3_–SCoM to CO_2_^23^. In other words, there is hydrogen exchange between water and the methyl group. Another line of evidence is that a small portion of methane is derived from dissolved inorganic carbon even when is cultivated solely on methanol or acetate, as shown by several previous reports.^43–45^ This is reflected in the observed variations of methane isotope signatures with δD_H2O_ in our D-spiked methylotrophic and acetoclastic methanogenesis experiments (Figure 3D, H and L), where there are offsets between the measured isotope values and the modeled parabolas that are expected for a “pure” exogenous combinatorial effect. To address this reversibility in a simple way, we assume that the final headspace is a mixture of methane produced from methanol or acetate and H_2_/CO_2_. This separates the gas into two components—a “pure” methylotrophic or acetoclastic endmember with a “pure” exogenous combinatorial effect, and a “pure” hydrogenotrophic endmember possessing the isotopic values of hydrogenotrophic methanogenesis. Here we assume the reactions from CO_2_ to CH_4_ in the methylotrophic and acetoclastic methanogenesis to have the same isotope effects as hydrogenotrophic methanogenesis, since they share the same reaction scheme (Figure 1). The isotopic values of the methylotrophic and acetoclastic endmembers are calculated from the measured isotopic values in the methylotrophic, acetoclastic and hydrogenotrophic samples by , assuming the proportion of methylotrophic or acetoclastic methanogenesis in the mixture is *r* (detailed in the Supporting Information). Variations of isotope values of the methylotrophic endmember with *r* are shown in Figure 3, which shows more prominent mixing effects at higher δD_H2O_. The *r* value used in the methylotrophic methanogenesis model is 0.985 (Table S3)—that is, 98.5% of total methane comes from methanol, with the remainder from inorganic carbon. We choose this value to match the prior observation that 1–2% of methane comes from inorganic carbon during the growth of on methanol.^43^ In contrast, the D-spiked acetoclastic methanogenesis experiment yields extremely positive Δ^12^CH_2_D_2_ values (Figure 3D,L) at higher δD_H2O_. By assigning a larger portion of methane from inorganic carbon (*r* = 0.92), these extremely positive values can be explained by the mixing of hydrogenotrophic and acetoclastic endmembers. After disentangling these two components, the remaining acetoclastic endmember fits the modeled results (Figure 3I-L).

Although the mixing effect can alter the observed isotope signatures in methylotrophic and acetoclastic methanogenesis, our results show that it is only significant at higher δD_H2O_ that is well above the natural range of δD_H2O_ (Figure 3). The degree of anticlumping from the combinatorial effect depends on the difference in the δD of water and the methylated compounds, which varies in nature depending on the environment as well. Nonetheless, the difference needs to be 2 orders of magnitude larger (nearly 3000‰, as opposed to −50 ‰) to erase the negative Δ^12^CH_2_D_2_ sourced from the exogenous combinatorial effect (Figure 3H,L). Therefore, it is unlikely that the natural variation of the source-material δD can eliminate the difference in Δ^12^CH_2_D_2_ between hydrogenotrophic methanogenesis and methanogenesis with methylated compounds.

Methoxydotrophic methanogenesis with TMB also possesses more negative Δ^12^CH_2_D_2_ than hydrogenotrophic methanogenesis, but less negative than acetoclastic and methylotrophic methanogenesis. This likely originates from a substantial input of methane (about one-third) from carbon dioxide through the Wood-Ljungdahl pathway.^4^ In this process, all hydrogen in the product methane comes from cellular hydrogen.^46^ Therefore, we assume it shows a similar signal as hydrogenotrophic methanogenesis. Since the microbial strain () used for the methoxydotrophic incubation is a hyperthermophile, we use the average isotope value of hydrogenotrophy under hyperthermophilic conditions as the endmember (Figure 4). Assuming 1/3 of the total methane comes from hydrogenotrophic methanogenesis,^4^ the “pure” methoxydotrophic methanogenesis endmember has a Δ^12^CH_2_D_2_ value of −52.5‰, similar to the “pure” methylotrophic and acetoclastic endmembers (Figure 4, Table S6).

**Figure 4:**
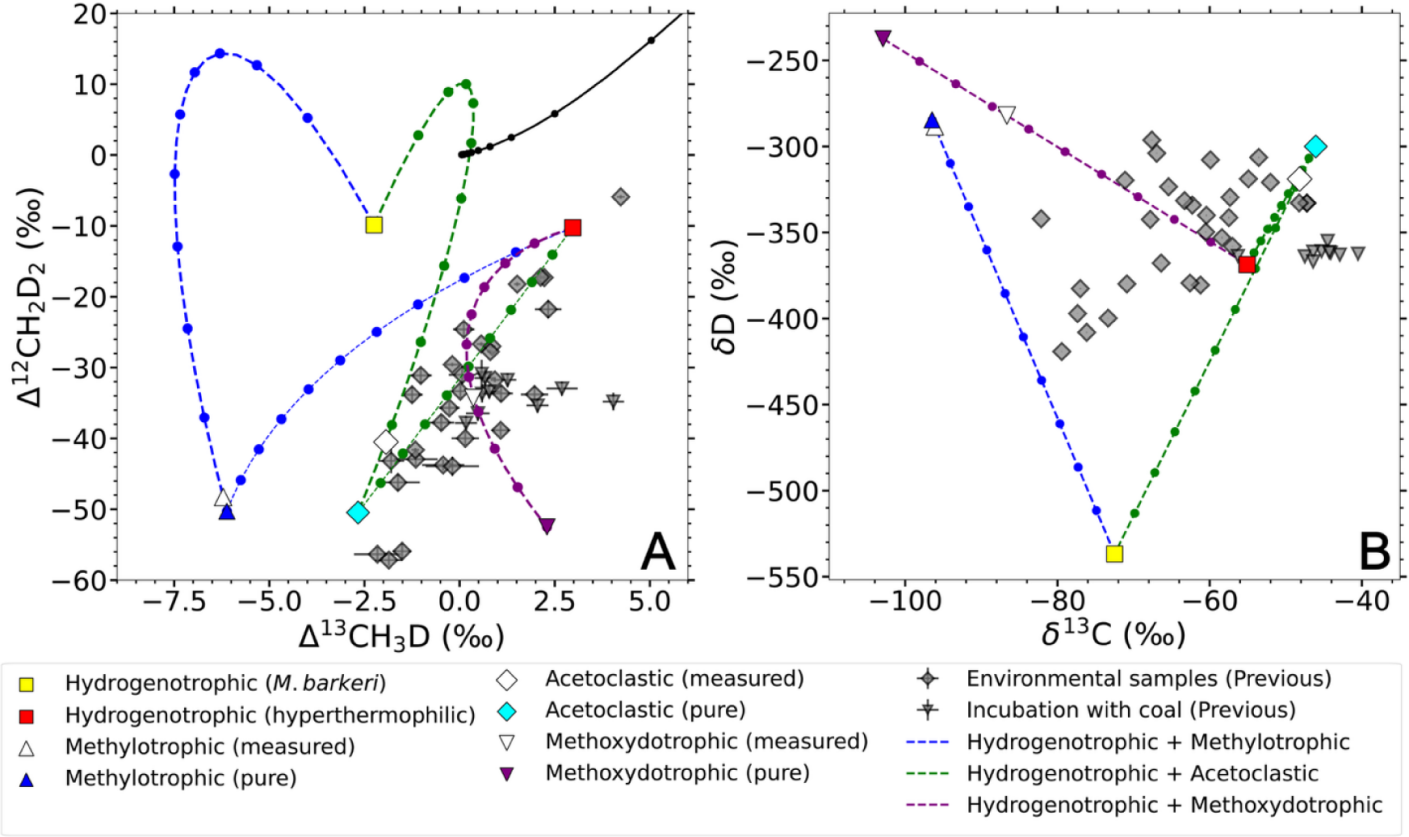
Mixing curves for (A) clumped and (B) bulk isotope between methanogenic pathways. The average “pure” hydrogenotrophic, methylotrophic, acetoclastic and methoxydotrophic methanogenesis endmembers derived from the mixing model are shown as colored points. The mixing curves between the hydrogenotrophic and methylotrophic, acetoclastic or methoxydotrophic endmembers are shown in dashed lines. Each point on the mixing curve represents a mixing ratio with 10% increment. The hollowed points are the average measured isotopic values in the experiments. The points in gray show the environmental or lab incubation data from the previous reports (Figure 2, Table S1).

### Methylotrophic and Acetoclastic Methanogenes Produce Markedly Different Δ^13^CH_3_D Values

Clumped isotopologue effects, represented by the four clumped isotopologue factors (^13CD^γ_p_, ^13CD^γ_s_, ^DD^γ_p_, ^DD^γ_s_ (described in detail in the Materials and Methods Section), also influence the modeled clumped isotope signatures (Figure S3). Previous reports have generally derived clumped isotopologue factors very close to unity in methanogenesis.^13,21–23^ Consistent with previous studies, the four clumped isotopologue factors derived in our model are very close to unity for acetoclastic methanogenesis, yielding 0.996, 0.999, 0.9975, and 0.999 for ^13CD^γ_p_, ^DD^γ_p_, ^13CD^γ_s_ and ^DD^γ_s_, respectively (Table S3).

In contrast, the best-fit values for the two primary clumped isotopologue factors significantly deviate from unity for methylotrophic methanogenesis (0.97 for both ^13CD^γ_p_ and ^DD^γ_p_, see Table S3), demonstrating a large deviation from the rule of geometric mean.^37^ In other words, the fractionation factors of the clumped isotopologues (^13CH3D^α and ^12CH2D2^α) are smaller than the products of the fractionation factors of the heavy isotopes in the molecules ^13^α^D^α and ^D^α^D^α, respectively). However, this is required to fit the isotopic data in this study, based on our analysis of the effects of ^13CD^γ_p_ and ^DD^γ_p_ on the modeled results (Figures S3G and S2L). Notably, we can reproduce the consistent decrease in Δ^13^CH_3_D with increasing δD_H2O_ by applying a smaller ^13CD^γ_p_ (Figures 3G, S3G). We attribute the relatively low values of the clumped isotopologue factors to different enzymes and reaction schemes in the methylotrophic methanogenesis pathway (Figure 1). Previous models are based on the reaction schemes of hydrogenotrophic methanogenesis.^13,21–23^ Methylotrophic methanogenesis, although sharing most of the reaction steps with hydrogenotrophic methanogenesis, uses a different set of enzymes to produce CH_3_–SCoM from CH_3_OH^47,48^ (Figure 1). Compared to acetoclastic methanogenesis, in which acetate is converted into CH_3_–H_4_MPT to form methane, the substrate is first converted to CH_3_–SCoM in methylotrophic methanogenesis (Figure 1). Furthermore, the enzymes used in these steps are different. Our data imply that the difference in enzymes and reaction schemes may yield different net ^13CD^γ_p_ and ^DD^γ_p_ values in methylotrophic methanogenesis compared to hydrogenotrophic and acetoclastic methanogenesis.

Our model predicts that increases in ^13CD^γ_p_ and ^13CD^γ_s_ causes increases in Δ^13^CH_3_D values (Figures S3G, S2O). This could be the reason for the increasing trend in the Δ^13^CH_3_D values across methylotrophic, acetoclastic and methoxydotrophic methanogenesis (Figure 5). Again, this pathway dependence of γ is potentially a result of different enzymes and reaction schemes involved in different methanogenic pathways (Figure 1).

**Figure 5:**
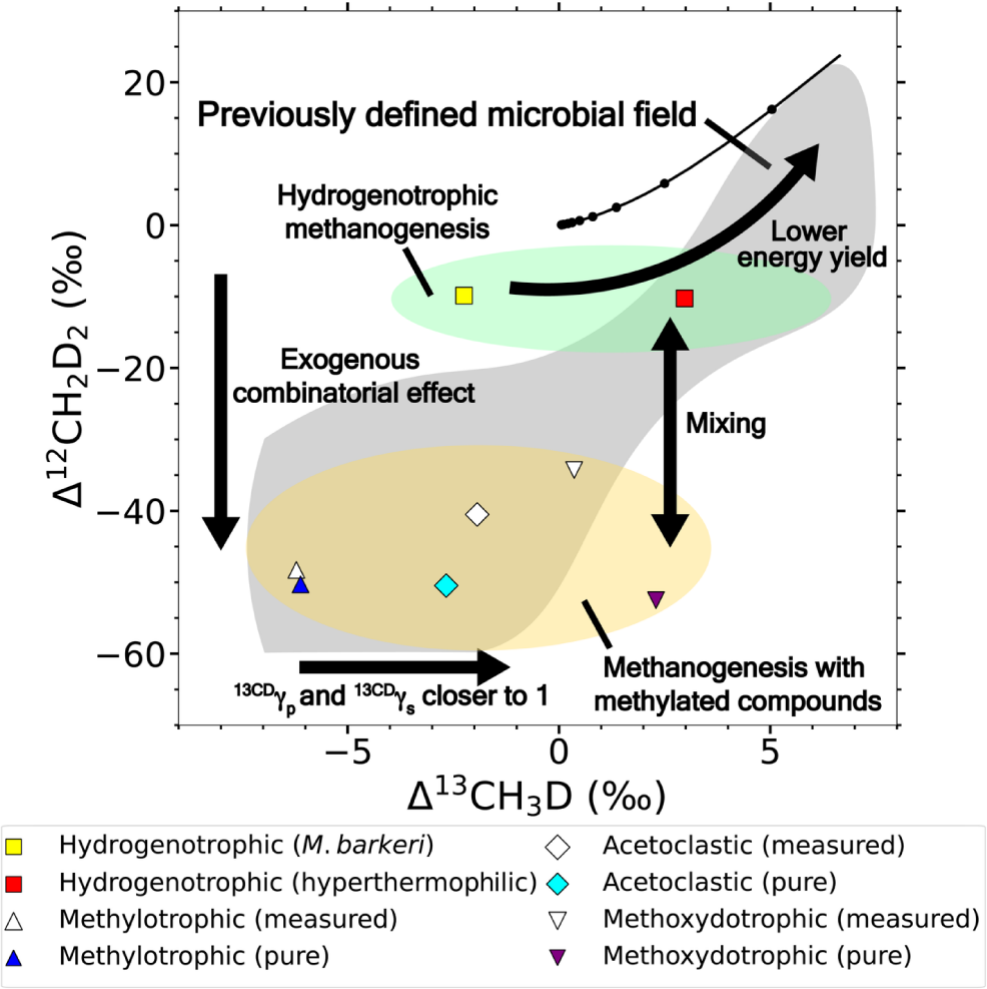
A summary of the influencing factors on the clumped isotope values of methanogenesis. The points on this figure show the average measured and “pure” clumped isotope values of each methanogenic pathway. The arrows show the processes that can influence the clumped isotope signatures, and the gray area shows the microbial methanogenesis field defined by the previous studies.^11,14,38^

### Variations in Methane Isotope Values within Hydrogenotrophic Methanogenesis

Within hydrogenotrophic methanogens, isotope signatures are different between mesophiles growing at 35 °C and hyperthermophiles growing from 65 to 80 °C (Figure 2). The isotopic values from hyperthermophilic strains broadly agree with the previous lab-cultured data,^14–17,23^ as shown in Figure 2. However, both the δD_CH4_ and Δ^13^CH_3_D values are different between mesophilic and hyperthermophilic strains in this study, despite similar δD values of water (Table S1). Moreover, the net hydrogen fractionation factor ^D^α_CH4‑H2O_ for mesophiles is 0.470, as represented by the slope of the regression line in Figure S2A. This value is below the previously reported range (0.56 to 0.86) for microbial hydrogenotrophic methanogenesis.^23,49–53^ In contrast, the ^D^α_CH4‑H2O_ for hyperthermophiles in this study ranges from 0.63 to 0.69 (Table S4), which falls within the previous range. We offer two possible explanations for this difference.

First, we point out that the thermodynamic drive available to the organisms is controlled by the bioenergetic environment provided by the concentrations of substrate, products and temperature. The net Gibbs free energy (ΔG_r_) set by environmental conditions influence the magnitudes of isotope fractionations within the methanogens.^21,22^ We estimated the net molar Gibbs free energy yield for the hydrogenotrophic methane-forming reaction in each experiment, based on the initial substrate concentration, the final methane production, and substrate consumption by stoichiometry (detailed in the Supporting Information). We used these net free energies as input to a model for isotope fractionations associated with each enzyme-mediated step of methane formation during hydrogenotrophic methanogenesis.^21^ The model predicts that δD_CH4_, Δ^13^CH_3_D, and Δ^12^CH_2_D_2_ values will decrease with increasing thermodynamic drive (more negative net ΔG_r_), while the δ^13^C_CH4_ value increases.^21^ The trends predicted by the model fit our experimental data (Figure S4), though the kinetic isotope effects of hydrogen addition steps retain large uncertainties.^21^ Interestingly, the hydrogen isotope fractionations between water and methane is not reproduced by the fractionation model without further parametrization, where we see an offset of ∼200 ‰ in ^2^ε_CH4‑H2O_ between the data by and the modeled values (Figure S4). This may be due to isotopic disequilibrium between intracellular H_2_ and H_2_O^21^.

Alternately, the key difference in the energy conservation approaches between the mesophilic strain () and hyperthermophilic strains (, , and ) may contribute to the different Δ^13^CH_3_D values. Similar observations were reported in a previous study, where consistently produced methane with negative Δ^13^CH_3_D at growth temperatures of 21 to 38 °C, whereas hyperthermophilic strains produce methane with positive Δ^13^CH_3_D at 30 to 80 °C^23^. While all the hydrogenotrophs we grow in this study share the same methanogenic pathway (Figure 1), during methanogenesis by , the reduction of ferredoxin is independent from the reduction of heterodisufide compound CoM–S–S-CoB. In contrast, during methanogenesis by the hyperthermophilic strains in this study, these two reactions are coupled by flavin-based electron bifurcation.^54^ Further studies on the isotopic fractionations for these two energy conservation processes, as well as the impact of thermodynamic drive on the net fractionation between substrates and products, will allow us to better interpret these observations.

### Variations in Methane Isotope Values within Methylotrophic Methanogenesis

Microbial methane produced from methanol shows no significant difference in clumped isotope composition despite different strains and growth temperatures, and our measurements agree with previous studies (Figure 2). This is likely due to the fact that the organisms in this study (, and ) share the same biochemical pathway to convert methanol to methane^55,56^ (Figure 1). One exception is performing methanogenesis with CH_3_OH + H_2_. The strain lacks enzymes to catalyze the upper branch of the reactions from CH_3_–SCoM to CO_2_^56,57^. Therefore, it potentially represents a “pure” methylotrophic methanogenesis endmember without the input of methane from inorganic carbon sources. For the other strains, only a negligible amount of methane is produced from inorganic carbon when methanol is the only provided substrate, as shown by the model (Figure 3). Therefore, it cannot significantly alter the “pure” methylotrophic methanogenesis signal without D-spiked water, thus all methylotrophic methanogenesis experiments with methanol and lab water demonstrate similar isotopic signatures (Figure 2).

In contrast, methanogenesis with TMA and TMA+H_2_ shows large differences in clumped isotope signatures, despite similarities in δD of methane (Figure 2). Methanogenesis with TMA demonstrates similar clumped isotope signatures as methanogenesis with CH_3_OH, whereas methanogenesis using TMA+H_2_ carries similar clumped isotope signatures as hydrogenotrophic methanogenesis (Figure 2A). This is contradictory to the expectation from the methanogenesis reaction schemes. In principle, growing on TMA+H_2_ should only produce methane from TMA,^55^ while growing on TMA should produce methane both from TMA and inorganic carbon.^6^ The reason behind this contradiction is not immediately clear from the available data in this study and our understanding of biochemistry (Figure 1). It is worth highlighting that the reactions from TMA to CH_3_–SCoM use a different set of methyltransferase (MT) enzymes than the reactions from CH_3_OH to CH_3_–SCoM.^47,48,57,58^ The fact that the conversions from tri-, di- and monomethylamine to CH_3_–SCoM are catalyzed by different sets of MT enzymes^55,57,59–61^ further complicates the analysis. Future experiments with D-spiked water and these substrates are necessary to understand the net primary and secondary isotope fractionation factors and test the effect of different enzymes on the isotope fractionation. Nonetheless, our study is the first report on the clumped isotope values of methylotrophic methanogenesis with TMA and opens more directions for future investigations.

### Implications for Differentiating Methane Sources in Natural Environments

This study provides experimental constrains on the isotopic fingerprints of multiple methanogenesis pathways. We estimate the “pure” methylotrophic, acetoclastic and methoxydotrophic endmembers from our natural abundance and D-spiked experiments (Figure 4A ,B). These results highlight the value of Δ^12^CH_2_D_2_ in distinguishing microbial methanogenesis via H_2_/CO_2_ versus methylated compounds, which stems from the exogenous combinatorial effect. To our knowledge, this is the first report of both Δ^13^CH_3_D and Δ^12^CH_2_D_2_ signatures of pure culture methanogenesis with acetate, TMB and TMA. Acetoclastic methanogenesis is a major contributor of methane production in freshwater sediments and anaerobic digesters.^3,8^ Although less common than hydrogenotrophic and acetoclastic methanogenesis, methanogenesis with TMB and TMA are also important in the methane cycling of some specific environments, for example, coal seams, hypersaline sediments and guts.^4,6,55^ Furthermore, the hydrogenotrophic methanogenesis by shows more negative δD and Δ^13^CH_3_D values, which are different from the values reported in previous studies (Figure 2). This expands the range of methane isotope values produced by microbes (Figure 5).

In many of our experiments, methane derived from inorganic carbon accompanies most of the methane producing metabolisms, due to the reversibility of the methanogenesis reactions,^62^ or the H_2_ production from the fermentation of methylated compounds.^43–45^ This means that the observed isotopic signatures of microbial methanogenesis will often be a mixture of at least two methanogenic pathways. In natural environments it has been shown that the dominant methanogenic pathway is determined by temperature^7,63^ and substrate availability.^6,8^ Although methanogenic reactions at low energy yield creates near-equilibrium isotopic signatures that are different from lab-cultured experiments and some environmental samples^12,13,20–22^ (Figure 5), this study focuses on methanogenesis under high energy yield, which is proposed to be very common in freshwater and terrestrial settings (Figure 4). Microbial methanogenesis operating far above the energy limit in nature usually demonstrates isotopic signals of mixing between the methanogenesis with H_2_/CO_2_ and methylated compounds,^39,64–68^ as shown in Figure 4. This demonstrates the potential of isotopic signals as tracers for the contributions of different methanogenic processes in nature. The incubation temperatures in this study are in the range of 35 to 80 °C, which are above the common temperature in most freshwater systems. However, comparing between the data in this study and previous studies,^14–16^ methanogenesis within the same reaction pathway shows relatively consistent isotopic signatures across a wide range of temperatures in lab-cultured experiments (Figure 2, Table S2). This implicates that the influence of temperature on the isotopic signals of methanogenesis is relatively small, whereas the enzymatic reaction pathways are the dominant controlling factors. Therefore, the results in this study may be extrapolated to lower temperatures when tracing the sources of methane in natural environments.

In this study, we provide a comprehensive look at the mass-18 isotopologues of methane as tracers of the major microbial methanogenesis pathways, and mechanistic explanations for the variations of isotope signatures within and between different methanogenic pathways grounded in biochemistry and chemical physics. This work enhances the utility of rare isotopologues of methane as tools for future studies on the sources and sinks of methane in both natural and engineered systems on Earth.

## Supplementary Material



## Data Availability

The Python, MATLAB scripts and data frames used in the analysis are on Github: https://github.com/Dart-Jarvis/Methanogenesis_est.

## Notes

The experimental details, materials, methods, measured isotope values, calculations of isotope fractionations, and modeling details, including Figure S1–S4, Table S1–S7, and Text S1–S7 (PDF)

## ⟁

Capture6, Berkeley, CA, 94703, United States

## ††

School of Biological Sciences, University of Nebraska-Lincoln, Lincoln, NE, 68588, United States.

## ‡‡

Department of Earth and Planetary Sciences, Northwestern University, Evanston, IL, 60208, United States.

## §§

Geology, Energy Please check: && Minerals (GEM) Science Center, U.S. Geological Survey, Reston, VA, 20192, United States.

## ▼

M.F. Deceased on May 11, 2022.
