# Supplementary material for: The Clumped Isotope Signatures of Multiple Methanogenic Metabolisms
Source: Environ Sci Technol. 2025 Jul 3;59(27):13798–810. doi: 10.1021/acs.est.5c03255 (PMC12269085; doi:10.1021/acs.est.5c03255)
Supplement: Supplementary file 1 [file es5c03255_si_001.pdf]

Supplementary Materials *for*:

The clumped isotope signatures of multiple methanogenic metabolisms

Jiawen Li[1], Jeanine L. Ash[2, †], Alec Cobban[1], Briana C. Kubik[3], Gabriella Rizzo[3, ‡], Mia Thompson[1], Laetitia Guibourdenche[4], Stefanie Berger[5], Kaycee Morra[6, §], Ying Lin[6], Elliott P. Mueller[7], Andrew L. Masterson[8, ¶], Rebekah Stein[9], Marilyn Fogel[6, #], Mark A. Torres[2], Xiahong Feng[1], James F. Holden[3], Anna Martini[10], Cornelia U. Welte[5], Mike S. M. Jetten[5], Edward D. Young[4], William D. Leavitt[1,11,12]*

Summary: This document has 39 pages, including 4 figures and 7 tables.

[1] Department of Earth Sciences, Dartmouth College, Hanover, NH, 03755, United States.

[2] Department of Earth, Environmental and Planetary Sciences, Rice University, Houston, TX, 77005, United States.

[3] Department of Microbiology, University of Massachusetts Amherst, Amherst, MA, 01003, United States.

[4] Department of Earth, Planetary, and Space Sciences, University of California, Los Angeles, Los Angeles, CA, 90095, United States.

[5] Department of Microbiology, RIBES, Radboud University, Nijmegen, 6525 XZ, the Netherlands.

[6] Department of Earth and Planetary Sciences, EDGE Institute, University of California, Riverside, Riverside, CA, 92521, United States.

[7] Division of Geological and Planetary Sciences, California Institute of Technology, Pasadena, CA, 91125, United States.

[8] Department of Earth and Planetary Sciences, Northwestern University, Evanston, IL, 60208, United States.

[9] Cooperative Programs for the Advancement of Earth System Science, University Corporation for Atmospheric Research, Boulder, CO, 80307, United States.

[10] Department of Geology, Amherst College, Amherst, MA, 01002, United States.

[11] Department of Geology & Geophysics, University of Utah, Salt Lake City, UT, 84112, United States.

[12] Department of Chemistry, Dartmouth College, Hanover, NH, 03755, United States.

Corresponding author:

Jiawen Li – Department of Earth Sciences, Dartmouth College, NH, United States.

Email address: jiawen.li.gr@dartmouth.edu

Present Addresses

[†] J.L.A.: Current address: Capture6, Berkeley, CA, 94703, United States.

[‡] G.R.: Current address: School of Biological Sciences, University of Nebraska-Lincoln, Lincoln, NE, 68588, United States.

[§] K.M.: Current address: Department of Earth and Planetary Sciences, Northwestern University, Evanston, IL, 60208, United States.

[¶] A.L.M.: Current address: Geology, Energy & Minerals (GEM) Science Center, U.S. Geological Survey, Reston, VA, 20192, United States.

Notes

[‡‡] M.F. Deceased on May 11, 2022.

Contents

1. **Supplementary Figures**

   Figure S1. Net carbon and hydrogen fractionation factors.

   Figure S2. Regression lines and equations between δD_{CH4} + 1000 and δD_{H2O} + 1000.

   Figure S3. Sensitivity analysis of the parameters on the isotopic signatures in the combinatorial effect model.

   Figure S4. The variation of isotopic signatures with Gibbs free energy of hydrogenotrophic methanogenesis.

2. **Supplementary Tables**

   Table S1. Reaction scheme

   Table S2. Compiled datasheet for isotopic data

   Table S3. Parameters of the combinatorial effect model

   Table S4. Net fractionation factors

   Table S5. Substrate isotope values

   Table S6. Average 'pure' endmember

   Table S7. Values of the model parameters used in the sensitivity test.

3. **Supplementary Texts**

   Text S1. Media recipes

   Text S2. Water and Substrate isotope measurements

   Text S3. A short description of the combinatorial effect

Text S4. Details in the model for the combinatorial effect

Text S5. Estimation of the Gibbs free energy yield in hydrogenotrophic methanogenesis

Text S6. Calculation of methanol consumption by stoichiometry

Text S7. Calculation of mixing

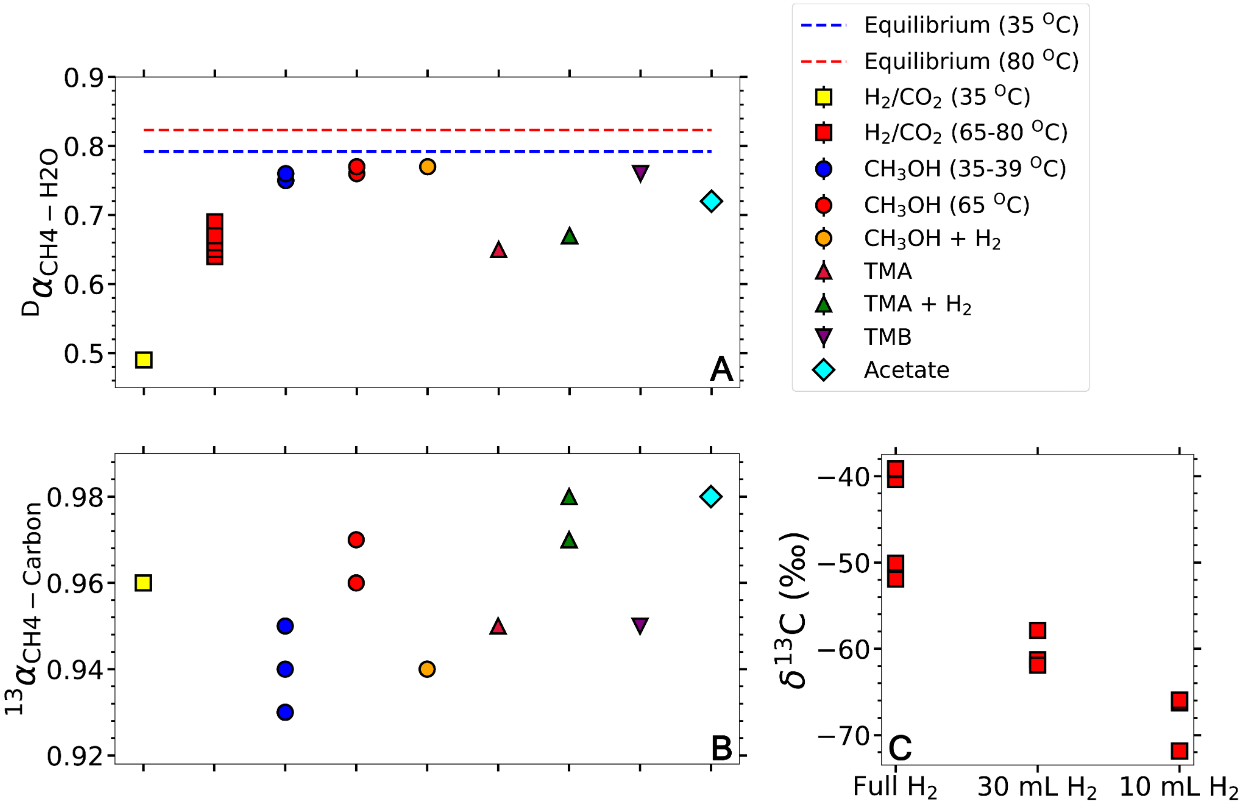

Figure S1. Net carbon and hydrogen fractionation factors with different substrates (Panel A and B), and bulk carbon isotope values of methane produced by hyperthermophilic hydrogenotrophic methanogens under different headspace H_2 (Panel C). The two dashed lines in panel A show the equilibrium isotope fractionation factors at 35 and 80 °C, based on the equation in the previous study[1]. Methane carbon isotope from hyperthermophile experiments is presented in the form of $\delta^{13}C$ relative to VPDB because $\delta^{13}C$ of DIC/CO_2 was not measured in these experiments.

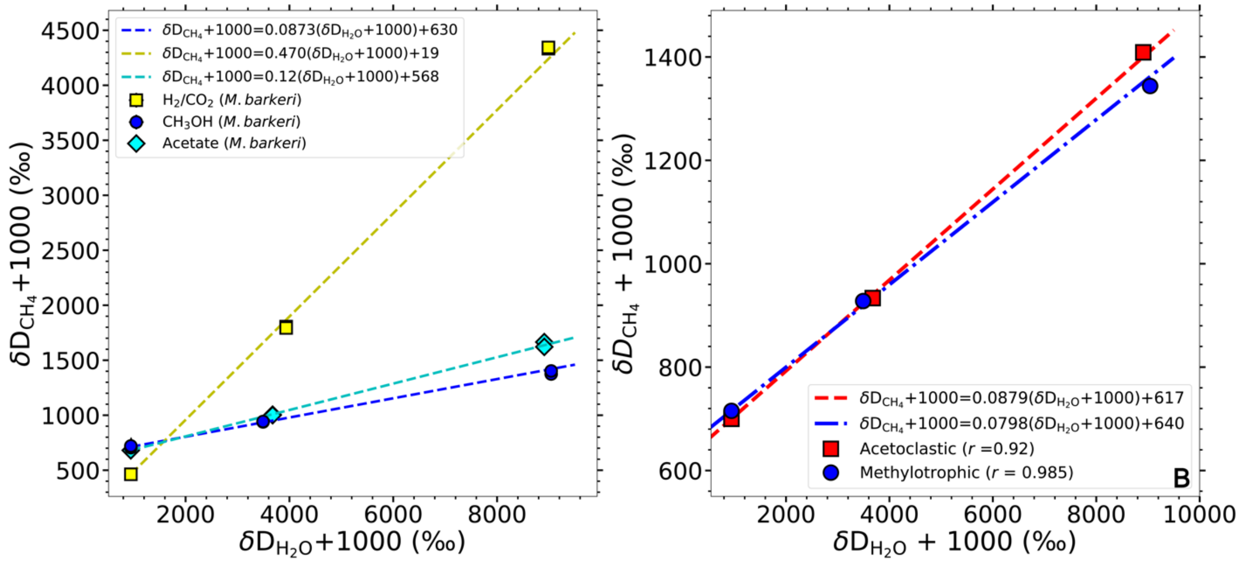

Figure S2. Linear regression lines and equations between $(\delta \mathrm{D_{CH4}} + 1000)$ **and** $(\delta \mathrm{D_{H2O}} + 1000)$ **for the (A) hydrogenotrophic (yellow squares), methylotrophic (blue circles) and acetoclastic (cyan diamonds) methanogenesis; (B) 'pure' methylotrophic (blue circles) and acetoclastic (red squares) endmembers.** δD of methane are expressed relative to VSMOW.

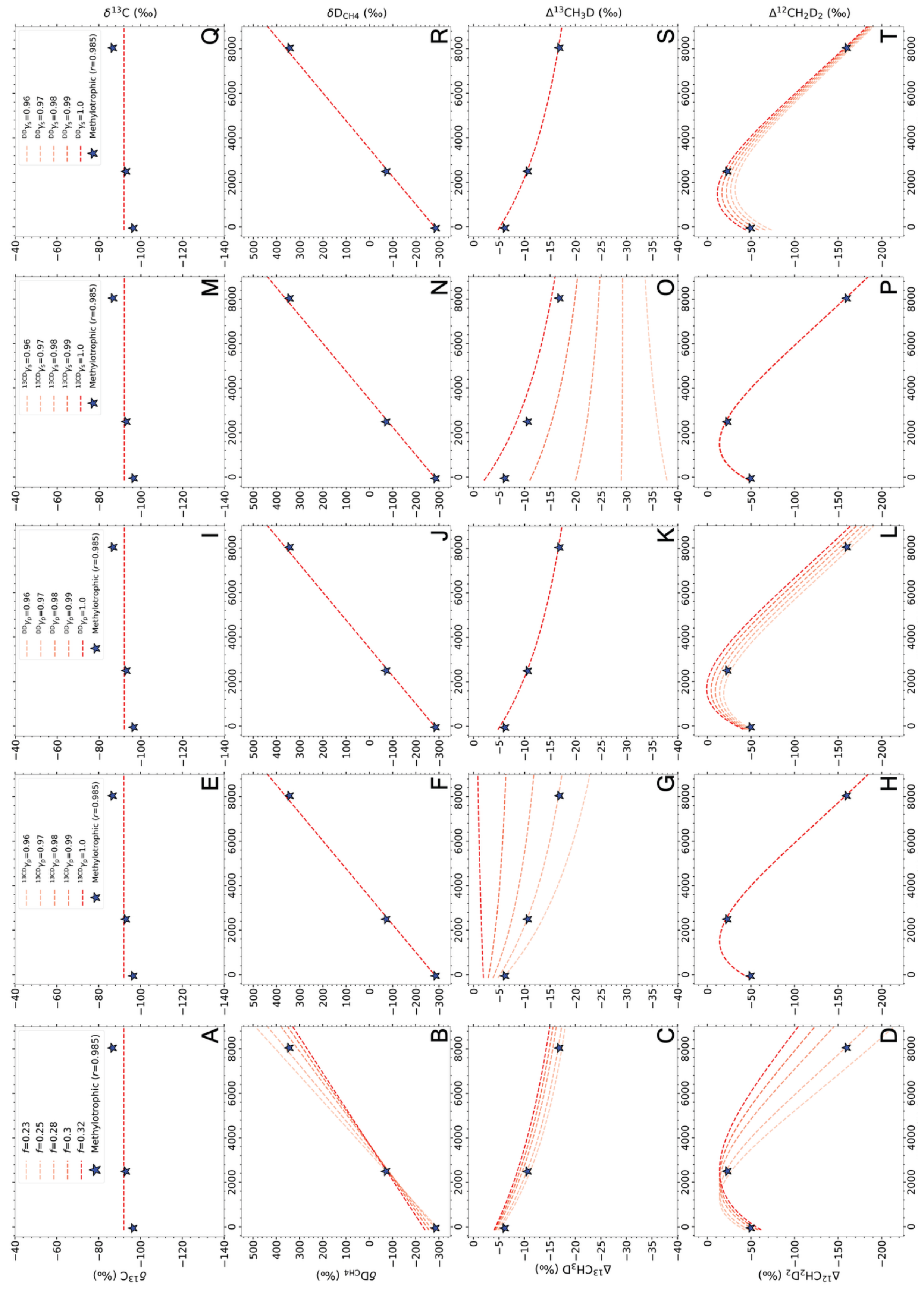
A
B
C
D
E
F
G
H
I
J
K
L
M
N
O
P
Q
R
S
T
f=0.23
f=0.25
f=0.28
f=0.3
f=0.32
Methylotrophic (r=0.985)
$^{13CD}\gamma_P$=0.96
$^{13CD}\gamma_P$=0.97
$^{13CD}\gamma_P$=0.98
$^{13CD}\gamma_P$=0.99
$^{13CD}\gamma_P$=1.0
$^{DD}\gamma_P$=0.96
$^{DD}\gamma_P$=0.97
$^{DD}\gamma_P$=0.98
$^{DD}\gamma_P$=0.99
$^{DD}\gamma_P$=1.0
$^{13CD}\gamma_S$=0.96
$^{13CD}\gamma_S$=0.97
$^{13CD}\gamma_S$=0.98
$^{13CD}\gamma_S$=0.99
$^{13CD}\gamma_S$=1.0
$^{DD}\gamma_S$=0.96
$^{DD}\gamma_S$=0.97
$^{DD}\gamma_S$=0.98
$^{DD}\gamma_S$=0.99
$^{DD}\gamma_S$=1.0
$\delta^{13}C$ (‰)
δD_{CH4} (‰)
$\Delta^{13}CH_3D$ (‰)
$\Delta^{12}CH_2D_2$ (‰)
δD_{H_2O} (‰)

Figure S3: Sensitivity analysis of the five parameters (f, $^{13CD}\gamma_p$, $^{DD}\gamma_p$, $^{13CD}\gamma_s$, $^{DD}\gamma_s$) on the isotopic signatures in the combinatorial effect model. Panels A-D, E-H, I-L, M-P and Q-T show the effects of f, $^{13CD}\gamma_p$, $^{DD}\gamma_p$, $^{13CD}\gamma_s$, $^{DD}\gamma_s$ on the isotopic signatures ($\delta^{13}C_{CH4}$, δD_{CH4}, $\Delta^{13}CH_3D$, $\Delta^{12}CH_2D_2$), respectively. Each parameter used in the sensitivity analysis is set at an initial value (see Table S3). At each test, one of the five parameters (f, $^{13CD}\gamma_p$, $^{DD}\gamma_p$, $^{13CD}\gamma_s$, $^{DD}\gamma_s$) changes within the values shown in the legends (also in Table S3). As a reference, the 'pure' methylotrophic methanogenesis endmembers are shown in stars. Bulk $\delta^{13}C$ and δD of methane are measured relative to VPDB and VSMOW, respectively.

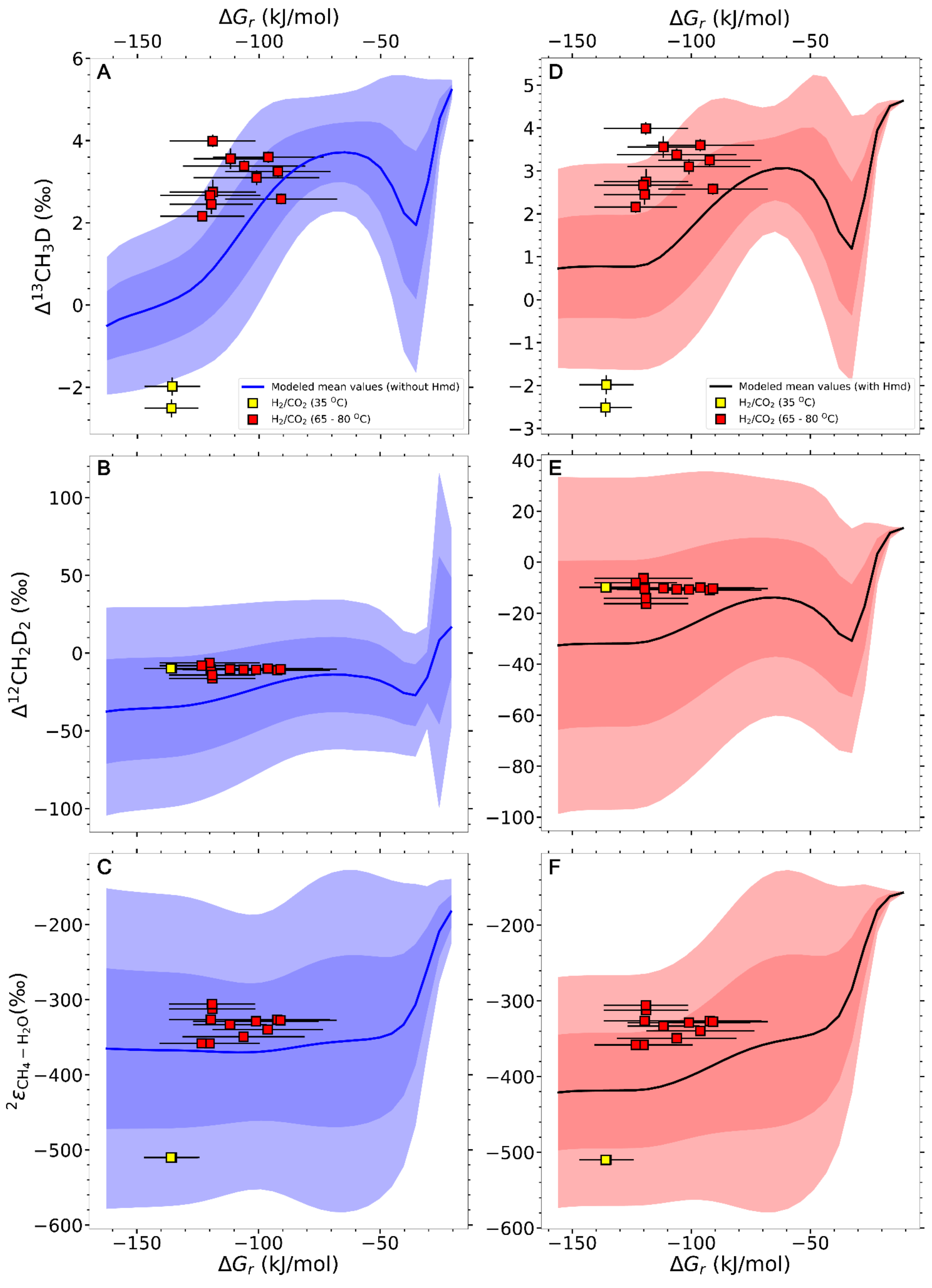

A
B
C
D
E
F
ΔG_r (kJ/mol)
−150
−100
−50
Δ13CH3D (‰)
Δ12CH2D2 (‰)
2ε CH4 − H2O (‰)
Modeled mean values (without Hmd)
Modeled mean values (with Hmd)
H2/CO2 (35 °C)
H2/CO2 (65 - 80 °C)

Figure S4: **The variation of isotopic signatures with Gibbs free energy (ΔG_r) of hydrogenotrophic methanogenesis.** Panels A-C are the two clumped isotope signatures ($\Delta^{13}CH_3D$, $\Delta^{12}CH_2D_2$) and hydrogen isotope fractionation between methane and water ($^2\varepsilon_{CH4-H2O}$) using the model with H_2-forming methylene tetrahydromethanopterin dehydrogenase (Hmd). Panels D-F are the modeled results without Hmd[2]. The deep and shallow colored areas mark one and two standard deviations of the results, respectively. The hydrogenotrophic methanogenesis data in this study are shown in colored squares. The bars show the range of ΔG_r over the course of the experiments. The model is adopted from the previous study[2], and the uncertainties of the kinetic isotope effects (KIE) at each step are set as 1.5 times of the original uncertainties.

Table S1. Reaction schemes and relative reaction rates. $^{13}\alpha$, $^{D}\alpha_p$, $^{D}\alpha_s$ are the fractionation factor for ^{13}C, and the primary and secondary fractionation factors for hydrogen, respectively. The bracketed variables are the abundances of methane and water isotopologues in the system.

Reactions*	Reaction rates	Eqn. number
**For ^{12}C species**		
$^{12}CH_3 - R + H_2O \rightarrow {}^{12}CH_4$	$r_1 = [^{12}CH_3R][H_2O]$	(1)
$^{12}CH_2D - R + H_2O \rightarrow {}^{12}CH_3D$	$r_2 = {}^{D}\alpha_s[^{12}CH_2DR][H_2O]$	(2)
$^{12}CHD_2 - R + H_2O \rightarrow {}^{12}CH_2D_2$	$r_3 = {}^{DD}\gamma_p({}^{D}\alpha_s{}^2)[^{12}CHD_2R][H_2O]$	(3)
$^{12}CH_3 - R + HDO \rightarrow {}^{12}CH_4$	$r_4 = ½ [HDO][^{12}CH_3R]$	(4)
$^{12}CH_3 - R + HDO \rightarrow {}^{12}CH_3D$	$r_5 = ½ {}^{D}\alpha_p[HDO][^{12}CH_3R]$	(5)
$^{12}CH_2D - R + HDO \rightarrow {}^{12}CH_3D$	$r_6 = ½ {}^{D}\alpha_s[HDO][^{12}CH_2DR]$	(6)
$^{12}CH_2D - R + HDO \rightarrow {}^{12}CH_2D_2$	$r_7 = ½ ({}^{DD}\gamma_p){}^{D}\alpha_p{}^{D}\alpha_s[HDO][^{12}CH_2DR]$	(7)
$^{12}CHD_2 - R + HDO \rightarrow {}^{12}CH_2D_2$	$r_8 = ½ ({}^{DD}\gamma_s)({}^{D}\alpha_s{}^2)[HDO][^{12}CHD_2R]$	(8)
$^{12}CH_3 - R + D_2O \rightarrow {}^{12}CH_3D$	$r_9 = {}^{D}\alpha_p[D_2O][^{12}CH_3R]$	(9)
$^{12}CH_2D - R + D_2O \rightarrow {}^{12}CH_2D_2$	$r_{10} = ({}^{DD}\gamma_p){}^{D}\alpha_p{}^{D}\alpha_s[D_2O][^{12}CH_2DR]$	(10)
**For ^{13}C species**		
$^{13}CH_3 - R + H_2O \rightarrow {}^{13}CH_4$	$r_{11} = {}^{13}\alpha[^{13}CH_3R][H_2O]$	(11)
$^{13}CH_2D - R + H_2O \rightarrow {}^{13}CH_3D$	$r_{12} = ({}^{13CD}\gamma_s){}^{13}\alpha{}^{D}\alpha_s[^{12}CH_2DR][H_2O]$	(12)
$^{13}CH_3 - R + HDO \rightarrow {}^{13}CH_4$	$r_{13} = ½ {}^{13}\alpha[HDO][^{13}CH_3R]$	(13)
$^{13}CH_3 - R + HDO \rightarrow {}^{13}CH_3D$	$r_{14} = ½ ({}^{13CD}\gamma_p){}^{13}\alpha{}^{D}\alpha_p[HDO][^{13}CH_3R]$	(14)
$^{13}CH_2D - R + HDO \rightarrow {}^{13}CH_3D$	$r_{15} = ½ ({}^{13CD}\gamma_s){}^{13}\alpha{}^{D}\alpha_s[HDO][^{13}CH_2DR]$	(15)
$^{13}CH_3 - R + D_2O \rightarrow {}^{13}CH_3D$	$r_{16} = ({}^{13CD}\gamma_p){}^{13}\alpha{}^{D}\alpha_p[D_2O][^{13}CH_3R]$	(16)

* In the list of reactions, CH_3R can be either CH_3OH or CH_3COOH.

† The reaction rates in this table are relative reaction rates, assuming the second-order rate constant for reaction 1 is unity.

Table S2. **A compilation of isotopic data of lab-cultured methanogenesis experiments in this study and previous studies.** All isotopic data are shown in ‰.

Strain	δD_{H2O}	1σ*	Substrate	Temp/°C	$\delta^{13}C_{CH4}$	1σ	δD_{CH4}	1σ	$\Delta^{13}CH_3D$	1σ	$\Delta^{12}CH_2D_2$	1σ	total CH4 (mmol)	Incubation Time (hours)	Reference
**Rice/Radboud**															
M. luminyensis	-50.3	0.1	CH_3OH + H_2	37	-97.31	0.005	-271.38	0.02	-2.51	0.15	-44.85	0.62	1513	216	this study
M. luminyensis	-50.3	0.1	CH_3OH + H_2	37	-97.96	0.004	-271.95	0.01	-2.15	0.15	-44.5	0.58	1613	216	this study
M. luminyensis	-50.3	0.1	TMA + H_2	37	-64.91	0.003	-359	0.02	-0.26	0.13	-17.86	0.64	1222	216	this study
M. luminyensis	-50.3	0.1	TMA + H_2	37	-69.64	0.005	-362.56	0.03	-0.48	0.21	-16.49	1.02	264	216	this study
M. luminyensis	-50.3	0.1	TMA + H_2	37	-67.95	0.005	-361.87	0.03	0.08	0.18	-16.55	0.94	618	216	this study
M. shengliensis	-50.3	0.1	CH_3OH	65	-79.11	0.003	-275.58	0.03	-4.17	0.14	-45.8	0.66	1671	55	this study
M. shengliensis	-50.3	0.1	CH_3OH	65	-72.15	0.003	-270.38	0.03	-5.51	0.12	-47.82	0.56	1794	55	this study
M. shengliensis	-50.3	0.1	CH_3OH	65	-66	0.005	-269.49	0.03	-3.22	0.21	-45.7	0.8	2069	55	this study
M. shengliensis	-50.3	0.1	TMB	65	-86.65	0.005	-282.04	0.02	0.36	0.21	-34.29	0.72	1064	128	this study
M. mazei	-50.3	0.1	CH_3OH	39	-108.64	0.004	-287.12	0.03	-3.51	0.16	-51.85	0.83	408	216	this study
M. mazei	-50.3	0.1	CH_3OH	39	-108.73	0.006	-287	0.03	-3.95	0.22	-48.1	1.04	339	216	this study
M. mazei	-50.3	0.1	TMA	39	-89.47	0.003	-379.52	0.02	-3.45	0.14	-31.29	0.79	1512	151	this study
**Dartmouth (substrate)**															
M. barkeri	-54.7	0.2	H_2+CO_2	35	-73.15	0.004	-536.75	0.03	-1.98	0.22	-9.88	0.98	93	2208	this study
M. barkeri	-54.7	0.2	H_2+CO_2	35	-71.83	0.01	-536.88	0.03	-2.51	0.22	-9.86	0.97	88	2208	this study
M. barkeri	-55	0.3	CH_3OH	35	-103.9	0.005	-295.19	0.02	-4.43	0.12	-47.65	0.68	136	2208	this study
M. barkeri	-55	0.3	CH_3OH	35	-98.5	0.005	-290.48	0.04	-9.24	0.19	-47.88	0.87	185	2208	this study
M. barkeri	-55	0.3	CH_3OH	35	-85.93	0.005	-279.4	0.02	-5.21	0.22	-49.48	0.75	355	2208	this study
M. barkeri	-56	0.4	Acetate	35	-47.77	0.003	-318.49	0.02	-1.96	0.24	-39.77	0.77	231	2208	this study
M. barkeri	-56	0.4	Acetate	35	-48.58	0.029	-319.38	0.02	-1.91	0.24	-41.24	0.73	223	2208	this study
**Dartmouth (water-spike/substrate)**															
M. barkeri	2937.2	3.3	H_2+CO_2	35	-75.52	0.005	806.05	0.05	-4.87	0.14	-16.25	0.43	81	2208	this study
M. barkeri	2937.2	3.3	H_2+CO_2	35	-74.6	0.012	793.24	0.04	-5.27	0.14	-15.51	0.45	72	2208	this study
M. barkeri	7988	6.9	H_2+CO_2	35	-74.14	0.004	3333.25	0.18	-4.57	0.13	-14.6	0.31	114	2208	this study
M. barkeri	7988	6.9	H_2+CO_2	35	-78.34	0.004	3344.29	0.17	-5.03	0.11	-18	0.39	52	2208	this study
M. barkeri	2491.9	2.8	CH_3OH	35	-95.23	0.004	-60.94	0.02	-10.39	0.13	-10.01	0.56	167	2208	this study
M. barkeri	2491.9	2.8	CH_3OH	35	-90.26	0.003	-57.29	0.02	-9.99	0.31	-11.61	0.57	176	2208	this study
M. barkeri	8041.9	6.7	CH_3OH	35	-93.7	0.007	374.73	0.03	-15.93	0.11	-95.99	0.45	184	2208	this study

M. barkeri	8041.9	6.7	CH_3OH	35	-79.27	0.004	402.66	0.03	-15.96	0.24	-68.16	0.55	352	2208	this study
M. barkeri	2676	4.1	Acetate	35	-53.66	0.004	5.39	0.02	-4.38	0.22	43.25	0.53	81.8	2208	this study
M. barkeri	2676	4.1	Acetate	35	-53.16	0.004	0.3	0.02	-4.65	0.16	39.67	0.62	106.1	2208	this study
M. barkeri	7909	7.9	Acetate	35	-54.09	0.004	665.18	0.05	-6.41	0.33	98.67	0.61	67.1	2208	this study
M. barkeri	7909	7.9	Acetate	35	-54.1	0.006	621.53	0.05	-7.08	0.29	80.56	0.54	62.3	2208	this study
**U. Mass**															
M. jannaschii	-52.9	0.2	H_2 (full)+CO_2	80	-51.93	0.009	-348.6	0.03	2.75	0.3	-16.28	0.82	323	3-5	this study
M. bathoardescens	-53.1	0.2	H_2 (full)+CO_2	80	-40.38	0.005	-362.18	0.03	2.45	0.24	-10.4	1.08	310	3-5	this study
M. thermolithotrophicus†	-53.5	0.1	H_2 (full)+CO_2	65	-39.65	0.005	-403.85	0.04	0.99	0.19	1.75	0.98	376	3-5	this study
M. thermolithotrophicus	-53.5	0.1	H_2 (full)+CO_2	65	-39.22	0.004	-392.46	0.03	2.67	0.13	-6.27	0.84	399	3-5	this study
M. thermolithotrophicus	-53.5	0.1	H_2 (full)+CO_2	65	-39.13	0.005	-392.41	0.03	2.16	0.13	-8.08	0.76	344	3-5	this study
M. jannaschii	-52.9	0.2	H_2 (full)+CO_2	80	-50.07	0.007	-342.57	0.03	3.99	0.15	-14.13	0.82	321	3-5	this study
M. jannaschii	-53	0.2	H_2 (30 mL) + CO_2	80	-61.26	0.008	-368.63	0.03	3.56	0.25	-10.25	1.1	137	5-8	this study
M. bathoardescens	-53.4	0.1	H_2 (30 mL) + CO_2	80	-57.87	0.005	-364.55	0.02	3.1	0.18	-10.73	0.78	194	5-8	this study
M. thermolithotrophicus	-52.6	0.3	H_2 (30 mL) + CO_2	65	-61.89	0.003	-383.53	0.02	3.38	0.14	-10.61	0.79	203	5-8	this study
M. jannaschii	-53	0.2	H_2 (10 mL) + CO_2	80	-71.84	0.004	-362.32	0.03	3.25	0.14	-11	0.96	63	10-15	this study
M. thermolithotrophicus	-52.3	0.2	H_2 (10 mL) + CO_2	65	-66.25	0.004	-374.26	0.03	3.6	0.14	-9.96	0.81	77	10-15	this study
M. bathoardescens	-53.8	0.1	H_2 (10 mL) + CO_2	80	-65.96	0.004	-363.71	0.03	2.58	0.13	-10.36	0.8	64	10-15	this study
**Previous work**															
M. acetivorans			CH_3OH	30	-32.77	0.007	-328.41	0.02	-3.88	0.2	-40.86	0.36			[3]
M. acetivorans			CH_3OH	30	-32.76	0.005	-328.45	0.02	-3.84	0.09	-43.24	0.31			[3]
M. barkeri			CH_3OH	30	-56.55	0.007	-340.18	0.02	-1.11	0.13	-34.33	0.35			[3]
M. thermolithotrophicus			H_2+CO_2	65	-49.36	0.011	-394.37	0.02	2.66	0.1	-19.44	0.3			[3]
M. aeolicus			H_2+CO_2	46	-53.22	0.007	-390.9	0.02	2.83	0.22	-17.34	0.34			[4]
M. aeolicus			H_2+CO_2	46	-51.45	0.003	-399.6	0.01	3.8	0.3	-16.94	0.3			[4]
M. barkeri			CH_3OH	37	-53.7	0.004	-250.1	0.01	-2.37	0.01	-54.49	0.36			[4]
M. barkeri			CH_3OH	37	-53.36	0.005	-249.88	0.01	-2.26	0.01	-55.34	0.34			[4]
M. acetivorans			CH_3OH	28	-29.16	0.005	-343.22	0.11	-4.2	0.28	-30	1.54			[5]

M. acetivorans	CH_3OH	28	-88.22	0.005	-372.49	0.09	-6.4	0.32	-38	2.61	5
M. acetivorans	CH_3OH	28	-29.17	0.005	-343.03	0.07	-3.8	0.28	-35	1.69	5
M. maripaludis	$H_2 + CO_2$	37	-50.53	0.003	-372.56	0.02	2.3	0.12	-12.11	0.39	4
P. stutzeri	MPn‡	37	-99.99	0.006	-299.49	0.04	0.02	0.13	-52.28	0.83	6
P. stutzeri	MPn	37	-100.15	0.01	-300.22	0.03	0.5	0.24	-51.11	0.88	6
**Previous environmental samples**§											
Chamber(wetland)			-62.28	0.005	-334.07	0.04	-1.8	0.345	-43.17	1.75	7
Chamber(wetland)			-53.56	0.025	-306.37	0.075	0.07	0.355	-31.02	1.305	7
Chamber(wetland)			-54.91	0.01	-318.82	0.025	1.08	0.23	-38.85	0.83	7
Chamber(wetland)			-52.02	0.01	-320.85	0.025	0.15	0.36	-39.99	0.915	7
Chamber(wetland)			-47.23	0.01	-332.88	0.025	-1.86	0.36	-57.16	0.805	7
bubbles			-57.33	0.045	-329.55	0.02	-0.43	0.555	-43.75	0.77	8
bubbles			-60.43	0.045	-340.01	0.045	-0.19	0.7	-43.92	1.35	8
bubbles			-57.47	0.045	-341.34	0.025	-1.15	0.57	-42.93	0.905	8
chamber			-58.39	0.045	-353.01	0.035	-1.62	0.575	-46.21	1.145	8
chamber			-47.23	0.045	-332.88	0.025	-2.16	0.615	-56.35	0.96	8
chamber			-48.23	0.05	-332.81	0.02	-1.51	0.245	-55.92	1.07	8
Qingnian lake			-60.51	0.01	-349.7	0.06	0.02	0.22	-33.33	0.94	9
Youyi lake			-66.96	0.01	-303.96	0.06	0.11	0.28	-24.6	1.01	9
Aiwan lake			-67.81	0.01	-342.41	0.06	-0.27	0.27	-35.69	0.92	9
Qingnian lake			-67.6	0	-296.3	0.1	1.97	0.36	-33.8	1.15	9
Youyi lake			-65.4	0	-323.4	0.1	-0.48	0.34	-37.76	1.05	9
Aiwan lake			-59.9	0	-307.8	0.1	1.09	0.31	-33.65	1.1	9
Rice paddies			-63.3	0	-331.5	0.1	-1.02	0.29	-31.11	1.07	9
Rice paddies			-57	0	-358.1	0.1	-1.25	0.28	-33.83	1.08	9
Goldstream hotspot			-77	0.02	-382.6	0.2	2.33	0.35	-21.76	1.24	10
Coal incubation			-47.5	-	-363.8	-	0.17	0.29	-37.81	0.99	11
Coal incubation			-56.3	-	-363.6	-	0.67	0.28	-31.47	1.27	11
Coal incubation			-44.5	-	-354.9	-	2.69	0.41	-32.95	0.99	11

Coal incubation	-45.3	-	-360.9	-	2.05	0.29	-35.35	0.98	11
Coal incubation	-44.1	-	-361.7	-	4.04	0.28	-34.8	1.23	11
Coal incubation	-42.9	-	-362.8	-	0.59	0.3	-33.68	1.26	11
Coal incubation	-46.4	-	-361.1	-	1.26	0.23	-31.73	0.94	11
Coal incubation	-40.5	-	-362.3	-	0.48	0.31	-36.45	1.06	11
Coal incubation	-44.3	-	-361.3	-	0.78	0.28	-33.37	0.96	11
Coal incubation	-46.4	-	-366.8	-	0.58	0.16	-30.87	1.96	11

* 1-s error of the measurements.

† The clumped isotope values of this data point are away from the other two replicates under the same condition and are close to the equilibrium values between 400 to 450 °C (Figure 2). This is potentially due to mishandling during the experiment, thus is excluded from the analysis.

‡ MPn: methylphosphonic acid.

§ Data from Arctic lakes in a previous study[12] is also included, but not shown in this table.

Table S3. Input parameters of the model for combinatorial effect. The values and uncertainties of the measured parameters are from the experimental data, and those for the fixed parameters are determined by the fraction of hydrogen from water (*f*), which is set at 0.25 and assigned an uncertainty of 0.01. The free parameters are assigned to fit the isotopic data of the samples. One set of input parameters is used for each model for the methylotrophic and acetoclastic methanogenesis data, respectively.

Input parameters	Methylotrophic	1-σ	Acetoclastic	1-σ
**Measured parameters**				
δD_{methyl} (‰)	-54.113	0.246	-159.7	1.9
$\delta^{13}C_{methyl}$ (‰)	-41.343	0.017	-32.2	0.05
$\Delta^{13}CH_2D_{methyl}$ (‰)	+0.842	0.352	0.0[ll]	0.300
$\Delta^{12}CD_2H_{methyl}$ (‰)	+6.255	3.776	0.0[ll]	3.000
**Fixed parameters**				
f	0.25	0.01	0.25	0.01
Slope*	0.0798	0.00006	0.0879	0.00008
Intercept*	640.373	0.090	616.925	0.097
$^{13}\alpha$†	0.9471	0.0052	0.9817	0.0035
$^{D}\alpha_p$‡	Slope/*f*	-	Slope/*f*	-
$^{D}\alpha_s$‡	Intercept/((1-*f*)(δD_{methyl} + 1000))	-	Intercept/((1-*f*)(δD_{methyl} + 1000))	-
**Free parameters**				
r	0.985	-	0.92	-
$^{13CD}\gamma_p$	0.9700	0.002	0.9960	0.002
$^{DD}\gamma_p$	0.9700	0.002	0.9990	0.002
$^{13CD}\gamma_s$	0.9970	0.002	0.9975	0.002
$^{DD}\gamma_s$	0.9950	0.002	0.9990	0.002
**Independent variable**				
δD_{H2O} (‰)§	[-150, 9000]	-	[-150, 9000]	-

* The values and 1-σ errors of the slopes and intercepts of the regression line are obtained from the least-square regression between $(\delta D_{CH4} + 1000)$ and $(\delta D_{H2O} + 1000)$ (see 'Details in the model for the combinatorial effect' for details), following the method in the previous study [13].

The regression is conducted on the ‘pure’ methylotrophic and acetoclastic endmember after eliminating the input from hydrogenotrophic methanogenesis (Figure S2).

† $^{13}\alpha$ is the carbon isotope fractionation factor between methane and substrates, after excluding the input from hydrogenotrophy.

‡ $^{D}\alpha_{p}$ and $^{D}\alpha_{s}$ are calculated from the slopes, intercepts and *f* using Equation S1. The uncertainties of $^{D}\alpha_{p}$ and $^{D}\alpha_{s}$ originate from the uncertainties in the slopes, intercepts and *f*. They are automatically propagated into the final results.

§ The isotope values are modeled against δD_{H2O}, which ranges from -150 to 9000 ‰, with a 50 ‰ increment (Figure 3).

|| The clumped isotope values of the methyl group in acetate are not measured in this study. Therefore, we set the values as 0 and assigned a similar uncertainty as methanol.

Table S4. Net fractionation factors.

Strain	Substrate	Temp	$^{2}\varepsilon_{CH4-H2O}$	1-σ	$^{D}\alpha_{CH4-H2O}$	1-σ	$^{13}\alpha_{CH4-Carbon}$	1-σ
**Rice/Radboud**								
M. luminyensis	$CH_3OH + H_2$	37 °C	-232.78	0.05	0.77	0.00005	0.94	0.0012
M. luminyensis	$CH_3OH + H_2$	37 °C	-233.37	0.04	0.77	0.00004	0.94	0.0012
M. luminyensis	$TMA + H_2$	37 °C	-325.04	0.04	0.67	0.00004	0.98	0.00034
M. luminyensis	$TMA + H_2$	37 °C	-328.79	0.05	0.67	0.00005	0.97	0.00034
M. luminyensis	$TMA + H_2$	37 °C	-328.06	0.04	0.67	0.00004	0.97	0.00034
M. shengliensis	CH_3OH	65 °C	-237.2	0.05	0.76	0.00005	0.96	0.00122
M. shengliensis	CH_3OH	65 °C	-231.72	0.05	0.77	0.00005	0.96	0.00123
M. shengliensis	CH_3OH	65 °C	-230.79	0.05	0.77	0.00005	0.97	0.00124
M. shengliensis	TMB	65 °C	-244.01	0.05	0.76	0.00005	0.95	0.0013
M. mazei	CH_3OH	39 °C	-249.35	0.05	0.75	0.00005	0.93	0.00118
M. mazei	CH_3OH	39 °C	-249.22	0.05	0.75	0.00005	0.93	0.00118
M. mazei	TMA	39 °C	-346.65	0.04	0.65	0.00004	0.95	0.00033
**Dartmouth, substrate**								
M. barkeri	H_2+CO_2	35 °C	-509.97	0.12	0.49	0.00012	0.96	0.00005
M. barkeri	H_2+CO_2	35 °C	-510.11	0.12	0.49	0.00012	0.96	0.00005
M. barkeri	CH_3OH	35 °C	-254.17	0.2	0.75	0.0002	0.93	0.00002
M. barkeri	CH_3OH	35 °C	-249.19	0.2	0.75	0.0002	0.94	0.00002
M. barkeri	CH_3OH	35 °C	-237.47	0.2	0.76	0.0002	0.95	0.00002
M. barkeri	Acetate	35 °C	-278.03	0.31	0.72	0.00031	0.98	0.00005
M. barkeri	Acetate	35 °C	-278.98	0.31	0.72	0.00031	0.98	0.00006

UMass

M. jannaschii	H_2 (full)+CO_2	80 °C	-312.21	0.14	0.69	0.00014
M. bathoardescens	H_2 (full)+CO_2	80 °C	-326.42	0.12	0.67	0.00012
M. thermolithotrophicus	H2 (full)+CO_2	65 °C	-370.17	0.1	0.63	0.0001
M. thermolithotrophicus	H_2 (full)+CO_2	65 °C	-358.14	0.09	0.64	0.00009
M. thermolithotrophicus	H_2 (full)+CO_2	65 °C	-358.09	0.09	0.64	0.00009
M. jannaschii	H_2 (full)+CO_2	80 °C	-305.84	0.14	0.69	0.00014
M. jannaschii	H_2 (30 mL)+CO_2	80 °C	-333.32	0.14	0.67	0.00014
M. bathoardescens	H_2 (30 mL)+CO_2	80 °C	-328.68	0.08	0.67	0.00008
M. thermolithotrophicus	H_2 (30 mL)+CO_2	65 °C	-349.27	0.18	0.65	0.00018
M. jannaschii	H_2 (10 mL)+CO_2	80 °C	-326.65	0.14	0.67	0.00014
M. thermolithotrophicus	H_2 (10 mL)+CO_2	65 °C	-339.72	0.11	0.66	0.00011
M. bathoardescens	H_2 (10 mL)+CO_2	80 °C	-327.5	0.11	0.67	0.00011

Table S5: Substrate isotope compositions.

Substrate	$\delta^{13}C$	1-σ*	δD†	1-σ	$\Delta^{13}CH_3D$	1-σ	$\Delta^{12}CH_2D_2$	1-σ
Radboud								
Methanol	-37.57	1.23	-65.03	0.32	-	-	-	-
TMA	-41.67	0.34	-109.73	0.16	-	-	-	-
TMB	-37.59	1.32	1.97	0.19	-	-	-	-
Dartmouth								
CO_2	-36.01	0.05	-	-	-	-	-	-
Methanol	-41.34	0.02	-54.11	0.25	0.842	0.352	6.26	3.78
Acetate	-32.2	0.05	-159.7	1.9	-	-	-	-

* 1 standard deviations of the measurements

† The δD values of methyl group/groups in the substrates.

Table S6. The average isotope values of the 'pure' methylotrophic, acetoclastic, and methoxydotrophic endmembers. All data are shown in ‰.

Substrates	*r*	$\delta^{13}C$	δD	$\Delta^{13}CH_3D$	$\Delta^{12}CH_2D_2$
**Methanol**					
-50 ‰ δD_{H2O}	0.985	-96.47	-284.57	-6.12	-49.48
+3000 ‰ δD_{H2O}	0.985	-93.01	-72.19	-10.62	-22.77
+8000 ‰ δD_{H2O}	0.985	-86.64	343.77	-16.81	-159.82
**Acetate**					
-50 ‰ δD_{H2O}	0.92	-46.06	-299.99	-2.67	-49.65
+3000 ‰ δD_{H2O}	0.92	-51.53	-66.44	-2.60	-10.73
+8000 ‰ δD_{H2O}	0.92	-52.17	408.97	-3.13	-198.75
**TMB**					
-50 ‰ δD_{H2O}	0.66	-102.92	-237.42	2.30	-52.50

Table S7. Values of the parameters used in the sensitivity test.

Input parameters	Initial values	Tested values*
**Fixed parameters**		
Slope	0.0798	-
Intercept	640.373	-
δD_{methyl} (‰)	-54.113	-
$\delta^{13}C_{methyl}$ (‰)	-41.343	-
$\Delta^{13}CH_2D_{methyl}$ (‰)	+0.842	-
$\Delta^{12}CD_2H_{methyl}$ (‰)	+6.255	-
$^{13}\alpha$	0.9471	-
r	0.985	-
δD_{H2O} (‰)†	[-150, 9000]	-
**Tested parameters**		
f	0.25	[0.23,0.25,0.28,0.3,0.32]
$^{D}\alpha_p$‡	Slope/f	-
$^{D}\alpha_s$‡	Intercept/((1-f)(δD_{methyl} + 1000))	-
$^{13CD}\gamma_p$	0.9700	[0.96,0.97,0.98,0.99,1.00]
$^{DD}\gamma_p$	0.9700	[0.96,0.97,0.98,0.99,1.00]
$^{13CD}\gamma_s$	0.9970	[0.96,0.97,0.98,0.99,1.00]
$^{DD}\gamma_s$	0.9950	[0.96,0.97,0.98,0.99,1.00]

* This shows the list of values used in the sensitivity test. During the test of each parameter, the values are chosen from the list of tested values, while the rest are set at the initial values.

† Similar to the model, the δD_{H2O} in each test ranges from -150 to 9000 ‰, with a 50 ‰ increment.

‡ $^{D}\alpha_p$ and $^{D}\alpha_s$ vary with f in the test.

Media recipes

Rice/Radboud

Methanosarcina media used for culturing of *M. mazei* and *M. luminyensis* were prepared with the reagents below and made anoxic by sparging with N_2/CO_2. The final pH was 6.8. 50 mL medium in serum bottles were inoculated with 2-10% of cultures along with ampicillin (final concentration 100µL), cysteine (1 mL of 0.75g/50 mL), Na_2S (1 mL of 0.75 g/50 mL), and either TMA (1 mL of 2.5 M solution) or methanol (1 mL of 7.5 M). Prior to incubating, bottles were filled with a H_2/CO_2 (80:20 v/v) mixture at 1 bar.

Reagent	Amount per L distilled H_2O
K_2HPO_4	0.348 g
KH_2PO_4	0.227 g
NH_4Cl	0.5 g
$MgSO_4$ x $7H_2O$	0.5 g
$CaCl_2$ x $2H_2O$	0.25 g
NaCl	2.25 g
$NaHCO_3$	0.85 g
Vitamin solution 1000x	1 mL
SL – 6 (altered for trace element content)*	1 mL
Yeast extract	2 g
Casiton	2 g
$NaCH_3COO$	1 g
Resazurin (1mg/mL)	1 mL

*1000 × trace element stock solution: 1.35 g/L $FeCl_2 \times 4\ H_2O$, 0.1 g/L $MnCl_2 \times 4\ H_2O$, 0.024 g/L $CoCl_2 \times 6\ H_2O$, 0.1 g/L $CaCl_2 \times 2\ H_2O$, 0.1 g/L $ZnCl_2$, 0.025 g/L $CuCl_2 \times 2\ H_2O$, 0.01 g/L H_3BO_3, 0.024 g/L $Na_2MoO_4 \times 2\ H_2O$, 0.22 g/L $NiCl_2 \times 6\ H_2O$, 0.017 g/L Na_2SeO_3, 0.004 g/L $Na_2WO_4 \times 2\ H_2O$, 12.8 g/L nitrilotriacetic acid.

Methermicoccus medium used for culturing *M. shengliensis* was prepared with the reagents below and made anoxic by sparging with N_2/CO_2. The final pH was 6.5. 50 mL medium in serum

bottles were inoculated with 2-10% of culture along with Na_2S (0.333 mL of 2.5 g/50 mL), cysteine (0.333 mL of 2.5 g/50 mL), Coenzyme M (100 µL of 3.125 g/ 25 mL) and either TMB (1.5 mL of 2.5 M) or methanol (1 mL of 7.5 M).

Reagent	Amount per L distilled H_2O
KCl	0.34 g
NH_4Cl	0.25 g
K_2HPO_4	0.20 g
NaCl	24 g
$MgCl_2$ x $6H_2O$	10.2 g
Yeast extract	2 g
Resazurin	1 mL of 0.1% solution
$NaHCO_3$	2.5 g
Vitamin solution 1000x	1 mL
SL – 6 (altered for trace element content)*	1 mL

*1000 × trace element stock solution: 1.35 g/L $FeCl_2 \times 4\ H_2O$, 0.1 g/L $MnCl_2 \times 4\ H_2O$, 0.024 g/L $CoCl_2 \times 6\ H_2O$, 0.1 g/L $CaCl_2 \times 2\ H_2O$, 0.1 g/L $ZnCl_2$, 0.025 g/L $CuCl_2 \times 2\ H_2O$, 0.01 g/L H_3BO_3, 0.024 g/L $Na_2MoO_4 \times 2\ H_2O$, 0.22 g/L $NiCl_2 \times 6\ H_2O$, 0.017 g/L Na_2SeO_3, 0.004 g/L $Na_2WO_4 \times 2\ H_2O$, 12.8 g/L nitrilotriacetic acid.

Dartmouth

HS medium

Mix A

Component	Amount	MW	final concentration
NaCl	23.4 g	58.45	400 mM
$NaHCO_3$	3.8 g	84.01	45 mM

KCl	1.0 g	74.56	13 mM
0.1% resazurin	1.0 mL	251.2	4 μM
Trace elements	10.0 mL	-	see formulation
Vitamin solution	10.0 mL	-	see formulation

Dissolve in ~750 mL distilled deionized H_2O. Boil for 10 minutes under a stream of N_2/CO_2 (80:20 v/v). Cool under flow, stopper and bring into an anaerobic chamber.

Mix B

Component	Amount	MW	final concentration
$MgCl_2.6H_2O$	11.0 g	203.31	54 mM
$CaCl_2.2H_2O$	0.3 g	147.02	2 mM

Dissolve in ~350 mL distilled deionized H_2O. Boil for 10 minutes under a stream of N_2/CO_2 (80:20 v/v). Cool under flow, stopper and bring into an anaerobic chamber.

Other Components

Component	Amount	MW	final concentration
1.0 M KH_2PO_4	5.0 mL	-	5 mM
NH_4Cl	1.0 g	53.49	19 mM
Cysteine.HCl	0.5 g	175.6	2.8 mM
0.2 M $Na_2S.9H_2O$	2.0 mL	------	0.4 mM
			Anaerobic, pH=6.8

Optional Components (all from sterile anaerobic stock solutions)

Component	Amount	MW	final concentration
100 % Methanol	5.0 mL/L	-	125 mM
5 M TMA-HCl	10.0 mL/L	-	50 mM
20 % yeast extract	10.0 mL/L	-	0.2 %
4.0 M Na acetate	10.0 mL/L	-	40 mM
10 % So Slurry	0.2 mL/20 mL	-	0.1 %
40 % glucose	0.8 mL/20 mL	-	1.0 %

Bring pre-weighed NH_4Cl, Cysteine.HCl, and $Na_2S.9H_2O$ into anaerobic chamber with Mixes A and B. Prepare sulfide solution with anaerobic water. Combine Mixes A and B and adjust volume to 1.0 liter with anaerobic water. Add NH_4Cl and Cysteine.HCl, allowing media to clear prior to addition of sulfide solution.

If desired, add other components such as methanol, glucose, sodium acetate or yeast extract from anaerobic stock solutions prior to dispensing in bottles. Alternatively, these can be added after autoclaving from sterile, anaerobic stock solutions.

Dispense medium into serum vials, stopper and bring out of the anaerobic chamber. Following volumes are recommended: for growth on H_2/CO_2 (80:20 v/v) 20 mL per 125 mL bottle, for all other medium 50 mL per 125 mL bottle, for Balch tubes 10 mL per tube.

Exchange gas four times for N_2/CO_2 (80:20 v/v) and pressurize to 5 psi. Autoclave 20 minutes at 121 ^oC. A precipitate will form upon autoclaving, but this will dissolve after the medium cools. Sterile medium should be stored in the anaerobic chamber until use.

For growth on H_2/CO_2 (80:20 v/v) pressurize to 25 psi after inoculation.

Modified Trace Elements Solution

Compound	Amt./Liter	MW	Conc.	Media Conc.
Nitrilotriacetic acid (Trisodium salt)	1.5 g	257.1	5.8 mM	58 µM
Fe(NH4)$_2$(SO$_4$)$_2$	0.8 g	392.1	2 mM	20 µM
Na_2SeO_3	0.2 g	172.9	1.1 mM	11 µM
$CoCl_2.6H_2O$	0.1 g	237.9	0.4 mM	4 µM
$MnSO_4.H_2O$	0.1 g	169	0.6 mM	6 µM
$Na_2MoO_4.2H_2O$	0.1 g	241.9	0.4 mM	4 µM
$Na_2WO_4.2H_2O$	0.1 g	329.9	0.3 mM	3 µM
$ZnSO_4.7H_2O$	0.1 g	287.5	0.3 mM	3 µM
$NiCl_2.6H_2O$	0.1 g	237.7	0.4 mM	4 µM
H_3BO_3	0.01 g	61.83	0.16 mM	1.6 µM
$CuSO_4.5H_2O$	0.01 g	249.7	40 µM	0.4 µM

Vitamin Solution

Compound	Amt./Liter	MW	Conc.	Media Conc.
p-Aminobenzoic acid	10 mg	137.1	73 µM	729 nM

Nicotinic acid	10 mg	123.1	81 µM	812 nM
Ca pantothenate	10 mg	238.3	42 µM	419 nM
Pyridoxine HCl	10 mg	205.6	49 µM	486 nM
Ribflavin	10 mg	376.4	27 µM	266 nM
Thiamine HCl	10 mg	337.3	30 µM	296 nM
Biotin	5 mg	244.3	20 µM	204 nM
Folic acid	5 mg	441.4	11 µM	113 nM
α-Lipoic acid	5 mg	206.3	24 µM	242 nM
Vitamin B12	5 mg	1355.4	3.7 µM	37 nM

Sterilize by filtration using a 0.22 micron filter

Store in the dark at 4 °C.

UMass

Each 60 mL serum bottle contains 25 mL of DSM 282 growth medium which contains (per liter): 780 mL of distilled deionized H_2O, 200 mL 5× salts (containing 150 g of NaCl, 20.5 g of $MgCl_2$•$6H_2O$, 17 g of $MgSO_4$•$7H_2O$, 1.65 g of KCl, 1.25 g of NH_4Cl, 0.7 g of $CaCl_2$•$2H_2O$, 0.7 g of K_2HPO_4), 10 mL of DSM 141 trace minerals solution, 10 mL of DSM 141 vitamin solution, 1 g of $NaHCO_3$, 1 g of $Na_2S_2O_3$, 0.10 ml of 0.1% (Na_2WO_4•$2H_2O$, 0.1% Na_2SeO_3) and 0.05 mL of 0.5% (w/v) resazurin solution. The medium was pH balanced to pH 6.00 ± 0.05 using 1 M HCl. Before inoculation, each bottle was reduced with 0.25 ml cysteine-HCl and 0.25 ml Na_2S•$9H_2O$.

Water and Substrate isotope measurements

The δD values of the water for incubations at Dartmouth College were measured at Dartmouth Stable Isotope Laboratory by the method in the previous study[14]. Water samples that were outside the range of the standards were diluted with a water of known isotopic compositions prior to the measurement using the method described in the previous study[6]. Water samples were then reduced to molecular hydrogen (H_2) by hot chromium at 850 °C; the isotope compositions of H_2 were measured by a dual-inlet isotope ratio mass spectrometer (IRMS, Thermo Delta Plus XL). The final results were reported as δD relative to VSMOW on the VSMOW-SLAP scale with uncertainties <0.5 ‰ (1σ).

Waters used during incubations at Radboud University were measured at Rice University on a Picarro Instruments L2310-*i* cavity ring-down spectrometer with an A0211 vaporization module and attached autosampler to determine $\delta^{18}O$ and δD. Samples are injected 8 times and run concurrently with 3 in-house standards (Elemental Microanalysis Zero Natural Isotope Water, Elemental Microanalysis Medium Natural Isotope Water, USGS RSIL-W-67400), a drift check of distilled in-house tap water (LT-3) and a quality-control check (USGS45). We report drift and memory corrected averages after van Geldern and Barth (van Geldern and Barth, 2012) of all injections normalized to the VSMOW-SLAP scale. Repeated measurements of USGS45 during this session had an external precision of 0.02 ‰ and 0.07 ‰ (2σ, n = 3) for $\delta^{18}O$ and δD respectively.

Bulk $\delta^{13}C$ values of CO_2 used in the incubations at Dartmouth College were measured following the protocols in a previous study[15] using a Gas Bench II coupled to a Delta V Plus isotope ratio mass spectrometer at the Northwestern University Stable Isotope Biogeochemistry Laboratory. Bulk hydrogen dD values were analyzed via a Gas Bench II. Two reference H_2 gases were standardized separately through repeated analysis of n-alkane reference materials supplied by Indiana University.

The isotopic values of the methyl group in the methanol used in the cultivation at Dartmouth College were analyzed in the Stolper Lab at UC Berkeley, following the previously published method[16]. Briefly, methanol was reacted in hydriodic acid (HI) to convert methoxyl groups on the methanol to iodomethane (CH_3I) and then chloromethane (CH_3Cl) for introduction to the Thermo Scientific 253 Ultra high-resolution dual-inlet isotope-ratio mass spectrometer. Final $\Delta^{13}CH_2D$, and $\Delta^{12}CHD_2$ values are all reported in a stochastic reference frame where 0 ‰ is equivalent to an infinite temperature for CH_3Cl, as determined by the previous study[16].

Sodium acetate solutions (50 μM in pure methanol) used in the incubations at Dartmouth College were analyzed on a heated electrospray ionization (HESI) Orbitrap QExactive HF (Thermo Fisher, Bremen, Germany) following the published protocol[17]. This method simultaneously measured the molecular average carbon isotope ratio of acetate and the hydrogen isotope ratio of acetate's methyl group. Sample isotope ratios were reported on the VPDB and VSMOW scales by comparison to a working standard of sodium acetate ($\delta^{13}C = -19.2‰$, $\delta D = -127‰$). The solutions were infused into the mass spectrometer using a Vanquish Horizons HPLC Split

Sampler Autosampler and a Vanquish Horizons Pump set to 5 μL/min with degassed LC-MS grade methanol as an eluent. An injection volume of 50 μL was carried by the eluent flow to the Orbitrap for a total of 10 acquisition minutes. At that time, the flow rate was increased to 30 μL/min to clear residual sample from the transfer lines. At 10.5 minutes, the flow rate was dropped again to 5 μL/min and 90 seconds later, the next injection began. Data acquisition included all 10 minutes but only integrated between 2 and 8 minutes to calculate isotope ratios. This was repeated to achieve bracketed, sample-standard comparisons (ABABABA, A = standard replicates, B = sample replicates). Acetate standard was diluted to match sample ion current and injected five times. Errors report the standard deviation of the $\delta^{13}C$ and δD of those replicates.

Bulk carbon and hydrogen isotopes of methanol (CH_3OH), trimethylamine (TMA) and 3,4,5-trimethoxybenzoate (TMB) used during incubations at Radboud University were analyzed at the EDGE Institute at UC Riverside. For $\delta^{13}C$ measurements, ~0.7 mg of each TMA/TMB sample and ~5.6 mg of each CH_3OH sample was weighed into a low permeability silver capsule and sealed with a liquid seal device then further wrapped in a more permeable tin capsule. These samples were combusted on a Costech ECS 4010 EA connected to a Thermo Scientific Delta V Advantage IRMS via a Conflo IV interface. One gelatin and three USGS glycine standards were analyzed concurrently for data correction. Within-run analytical precision was ~0.4‰. For δD measurements, ~1 mg of each sample was weighed into a silver capsule and pyrolyzed on a Thermo Scientific high temperature EA (TC/EA) connected to a Thermo Scientific Delta V Plus IRMS via a Conflo IV interface. Five standards of chicken and turkey feathers, stearic acid,

mineral oil, and pump oil were analyzed concurrently to form a regression for data correction. Within-run analytical precision was ~4‰.

A short description of the combinatorial effect

The stochastic distribution of isotopologues is calculated based on bulk isotope ratios assuming, by necessity, that the arithmetic mean of the D/H ratios comprising the molecules applies. However, where more than one D/H ratio contributes to the hydrogen pool for the molecules, the appropriate reference to calculate the stochastic distribution would be the geometric mean of these different ratios. The geometric mean will always be less than the arithmetic mean, meaning that the calculated stochastic ratios will be overestimated and the resulting Δ value underestimated.

Details in the model for the combinatorial effect

In the model for the combinatorial effect, we utilized the reaction schemes shown in Table S1. For simplicity, the reactions that produce triply substituted or heavier clumped isotopologues (e.g., $^{12}CHD_3$ and $^{13}CH_2D_2$) are not considered. Since these isotopologues are of very low abundance compared with lighter isotopologues, ruling them out will not cause a significant change in the modeled results for the isotopologues of interest. We categorize the parameters in the model into three parts. The measured parameters are determined experimentally, including the isotope values of the substrate methyl groups. The initial compositions of each isotopologues (bracketed values in Table S1) are calculated based on the measured isotope values of water and

substrates. The fixed parameters consist of the fraction of hydrogen from water (*f*), and a series of parameters that depend on *f* (Table S3). The free parameters include the mixing ratio *r*, and four clumped isotopologues factors $^{13CD}\gamma_p$, $^{13CD}\gamma_s$, $^{DD}\gamma_p$, $^{DD}\gamma_s$. The isotopic signatures of the 'pure' endmembers are determined by *r*.

In methylotrophic and acetoclastic methanogenesis, the hydrogen atoms in the product methane molecules come from two sources – water and methyl groups of the substrates. The mass balance of hydrogen gives the following equation:

$$\delta D_{CH4} + 1000 \ = \ ^{D}\alpha_s(1-f)(\delta D_{CH3} + 1000) +^{D} \alpha_p f(\delta D_{H2O} + 1000) \tag{S1}$$

Where $^{D}\alpha_p$ and $^{D}\alpha_s$ are primary and secondary fractionation factors. Primary fractionation represents the D/H fractionation during the addition of hydrogen to the methyl group, while secondary fractionation represents the D/H fractionation between the methyl group and product methane. *f* is the fraction of hydrogen in methane molecules that comes from water. To simplify the model, we set *f* at 0.25 in the model, meaning one out of four hydrogen atoms in the product methane molecule comes from water. This represents the methylotrophic or acetoclastic endmember with a 'pure' exogenous combinatorial effect (i.e. no hydrogen exchange between the methyl group and water). Following this assumption, we treat the headspace methane as a mixture derived from two sources – inorganic carbon (due to the reversibility of the oxidative branch of the methanogenesis) and methanol or acetate (methylotrophic or acetoclastic with exogenous combinatorial effect), and denote the portion of methylotrophic or acetoclastic endmembers in the mixture as *r*. When *f* is fixed to 0.25, the shape of parabola does not change by a lot with varying clumped isotopologue factors, as shown by Figure S3D, 2H, 2L, 2P and 2T.

Therefore, we first set the value of *r* to match the modeled parabola to eliminate the inputs methane from inorganic carbon and derive the isotopic values of the 'pure' methylotrophic and acetoclastic endmembers. We then obtained values of $^{\mathrm{D}}\alpha_{\mathrm{p}} f$ and $^{\mathrm{D}}\alpha_{\mathrm{s}}(1-f)(\delta \mathrm{D}_{\mathrm{CH3}}+1000)$ from the slopes and intercepts of the weighted least-square linear regression between $(\delta \mathrm{D}_{\mathrm{CH4}}+1000)$ and $(\delta \mathrm{D}_{\mathrm{H2O}}+1000)$ on the 'pure' methylotrophic and acetoclastic methanogenesis endmembers (Figure S2) . We calculated $^{\mathrm{D}}\alpha_{\mathrm{p}}$ and $^{\mathrm{D}}\alpha_{\mathrm{s}}$ by the slopes, intercepts and *f*, using eqn. S1, and the carbon isotope fractionation factors from the isotope data of the 'pure' methylotrophic and acetoclastic methanogenesis. Lastly, we tuned the four clumped isotopologues factors to get the best-fit values for the experimental data, using the reaction scheme in Table S1. The values and 1-σ uncertainties of all input parameters are listed in Table S3.

The observed trend of decreasing $\Delta^{13}CH_3D$ with increasing δD_{H2O} in methylotrophic methanogenesis (Figure 3C) contradicts with the expectation from a combinatorial effect. We rule out several other potential reasons for the observed trend. First, mixing has a minor effect on this trend, as shown in Figure 3G. Second, by stoichiometry, at most 38 % of methanol is consumed throughout the experiment (see the section 'Calculation of methanol consumption by stoichiometry'), thus a closed-system effect is unlikely to dominate. Moreover, the closed-system effect would drive the net fractionation factors towards unity, which is counter to our observations of the carbon isotope fractionation in methylotrophic methanogenesis (Figure S1, Table S4). Therefore, a large deviation in $^{13CD}\gamma_{p}$ from unity is the most plausible explanation for these data. It is worth highlighting that the largest δD_{H2O} used in the previous study on the

clumped isotope signatures of pure-culture methanogenesis is +335 ‰[18], much smaller than the δD_{H2O} used in this study (Table S1). From our model, a δD_{H2O} of +335 ‰ only produces about a 1 ‰ offset in $\Delta^{13}CH_3D$ from lab water with a δD_{H2O} of -50 ‰, which is within the external uncertainties between biological replicates.

Estimation of ΔG_r in hydrogenotrophic methanogenesis

The net Gibbs free energy yields (ΔG_r) and the corresponding isotopic signatures in hydrogenotrophic methanogenesis experiments are estimated following the model described in the previous study[2]. The net Gibbs free energy is calculated using the standard state Gibbs free energy (ΔG_0), as well as dissolved concentrations of H_2, CO_2 and CH_4:

$$\Delta G_r = \Delta G_0 + \mathrm{RTln}\frac{[CH_4]}{[CO_2][H_2]^4} \tag{S2}$$

Where the bracketed values are dissolved gas concentrations in mol/L. The standard Gibbs free energy at the given temperature is calculated from the Van't Hoff equation, following the previous literature[19]. The dissolved H_2, CO_2 and CH_4 concentrations in the ΔG_r calculations are obtained from Henry's law, using the headspace gas concentrations. The Henry's law constants for H_2, CO_2 and CH_4 are 7.5, 1.5 and 3.3 μmol/(m^3*Pa), respectively[20]. The initial H_2, CO_2 partial pressures are calculated from the initial growth conditions described in the Materials and Methods Section, while the initial CH_4 is set at a very low level (1/1000 of the initial CO_2 partial pressures). We estimated the partial pressures of the gasses at the end of the experiments by calculating the H_2 and CO_2 consumptions from CH_4 production, following the stoichiometry of the methanogenic reaction:

$$4H_2 + CO_2 \longrightarrow CH_4 + 2H_2O. \quad \text{(S3)}$$

We ran the model developed by Gropp et al.[2] with $\delta^{13}C$ and δD values of the substrates used in this study (-36.01 and -50.00 ‰, respectively). We also assigned larger uncertainties (1.5 times the original value) for the kinetic isotope effects of each reaction step in the model, since large uncertainties remain in those values[2]. The modeled results, both with and without Hmd activities, are shown in Figure S4.

Calculation of methanol consumption by stoichiometry

In methylotrophic methanogenesis, methanol is disproportionated into CH_4 and CO_2:

$$4CH_3OH \longrightarrow 3CH_4 + CO_2 + 2H_2O \quad \text{(S4)}$$

Therefore, we can calculate the consumption of methanol from the production of methane (Table S2) by stoichiometry. The consumption of methanol during the cultivation at Dartmouth College ranges from 181 to 473 µmol, corresponding to 14 to 38 % of the initial methanol (1250 µmol of initial methanol).

Calculation of mixing

The mixing between the hydrogenotrophic and acetoclastic/methylotrophic endmembers are calculated using the ratios of the isotopes or isotopologues. For bulk isotopes ($\delta^{13}C$ and δD), the δ values are first converted to fractional abundance (F):

$$^{13}F = \frac{^{13}R}{1+^{13}R} \quad \text{(S5)}$$

$$^{2}F = \frac{^{2}R}{1+^{2}R} \quad \text{(S6)}$$

Where R is the ratio of heavy to light isotopes (D/H or $^{13}C/^{12}C$) derived from the δ values. For the clumped isotopologues ($\Delta^{13}CH_3D$ and $\Delta^{12}CH_2D_2$), the absolute abundances of isotopologues that are measured on the instrument are used, which are calculated from the absolute abundances of isotopologues in the reference gas on Panorama[21]. The mixing effect is then calculated as:

$$F_{CH3-R} = \frac{F_{measured} - F_{H2/CO2}(1-r)}{r} \quad \text{(S7)}$$

Or

$$\mathrm{X_{CH3-R}} = \frac{\mathrm{X_{measured} - X_{H2/CO2}(1-r)}}{\mathrm{r}} \quad \text{(S8)}$$

Where “CH3-R”, “measured”, and “H2/CO2” in the subscripts denote the ‘pure’ methylotrophic or acetoclastic methanogenesis, measured samples, and hydrogenotrophic methanogenesis by *M. barkeri*, respectively. *F* and *X* are the fractional or absolute abundances of heavy isotopes or clumped isotopologues. *r* is the portion of methane derived from the methylotrophic or acetoclastic methanogenesis in the mixture. The calculated fractional abundances or absolute abundances are then converted to $\delta^{13}C$, δD, $\Delta^{13}CH_3D$ and $\Delta^{12}CH_2D_2$. Specifically, the stochastic distributions are calculated from the random distribution of isotopes[22]. The derived ‘pure’ endmembers are listed in Table S6.

References

(1) Horibe, Y.; Craig, H. DH Fractionation in the System Methane-Hydrogen-Water. *Geochim. Cosmochim. Acta* **1995**, *59* (24), 5209–5217. https://doi.org/10.1016/0016-7037(95)00391-6.

(2) Gropp, J.; Jin, Q.; Halevy, I. Controls on the Isotopic Composition of Microbial Methane. *Sci. Adv.* **2022**, *8* (14), eabm5713.

(3) Young, E. D.; Kohl, I. E.; Lollar, B. S.; Etiope, G.; Rumble, D.; Li (李姝宁), S.; Haghnegahdar, M. A.; Schauble, E. A.; McCain, K. A.; Foustoukos, D. I.; Sutclife, C.; Warr, O.; Ballentine, C. J.; Onstott, T. C.; Hosgormez, H.; Neubeck, A.; Marques, J. M.; Pérez-Rodríguez, I.; Rowe, A. R.; LaRowe, D. E.; Magnabosco, C.; Yeung, L. Y.; Ash, J. L.; Bryndzia, L. T. The Relative Abundances of Resolved l2CH2D2 and 13CH3D and Mechanisms Controlling Isotopic Bond Ordering in Abiotic and Biotic Methane Gases. *Geochim. Cosmochim. Acta* **2017**, *203*, 235–264. https://doi.org/10.1016/j.gca.2016.12.041.

(4) Giunta, T.; Young, E. D.; Warr, O.; Kohl, I.; Ash, J. L.; Martini, A.; Mundle, S. O. C.; Rumble, D.; Pérez-Rodríguez, I.; Wasley, M.; LaRowe, D. E.; Gilbert, A.; Sherwood Lollar, B. Methane Sources and Sinks in Continental Sedimentary Systems: New Insights from Paired Clumped Isotopologues 13CH3D and 12CH2D2. *Geochim. Cosmochim. Acta* **2019**, *245*, 327–351. https://doi.org/10.1016/j.gca.2018.10.030.

(5) Douglas, P. M.; Gonzalez Moguel, R.; Walter Anthony, K. M.; Wik, M.; Crill, P. M.; Dawson, K. S.; Smith, D. A.; Yanay, E.; Lloyd, M. K.; Stolper, D. A. Clumped Isotopes Link Older Carbon Substrates with Slower Rates of Methanogenesis in Northern Lakes. *Geophys. Res. Lett.* **2020**, *47* (6), e2019GL086756.

(6) Taenzer, L.; Labidi, J.; Masterson, A. L.; Feng, X.; Rumble III, D.; Young, E. D.; Leavitt, W. D. Low Δ12CH2D2 Values in Microbialgenic Methane Result from Combinatorial Isotope Effects. *Geochim. Cosmochim. Acta* **2020**, *285*, 225–236.

(7) Haghnegahdar, M. A.; Sun, J.; Hultquist, N.; Hamovit, N. D.; Kitchen, N.; Eiler, J.; Ono, S.; Yarwood, S. A.; Kaufman, A. J.; Dickerson, R. R.; Bouyon, A.; Magen, C.; Farquhar, J. Tracing Sources of Atmospheric Methane Using Clumped Isotopes. *Proc. Natl. Acad. Sci.* **2023**, *120* (47), e2305574120. https://doi.org/10.1073/pnas.2305574120.

(8) Haghnegahdar, M. A.; Hultquist, N.; Hamovit, N. D.; Yarwood, S. A.; Bouyon, A.; Kaufman, A. J.; Sun, J.; Magen, C.; Farquhar, J. A Better Understanding of Atmospheric Methane Sources Using $^{13}CH_3D$ and $^{12}CH_2D_2$ Clumped Isotopes. *J. Geophys. Res. Biogeosciences* **2024**, *129* (11), e2024JG008172. https://doi.org/10.1029/2024JG008172.

(9) Wang, X.; Liu, C.-Q.; Yi, Y.; Zeng, M.; Li, S.-L.; Niu, X. Machine Learning Predicts the Methane Clumped Isotopologue ($^{12}CH_2D_2$) Distributions Constrain Biogeochemical Processes and Estimates the Potential Budget. *Environ. Sci. Technol.* **2023**, *57* (46), 17876–17888. https://doi.org/10.1021/acs.est.3c00184.

(10) Shuai, Y.; Xie, H.; Zhang, S.; Zhang, Y.; Eiler, J. M. Recognizing the Pathways of Microbial Methanogenesis through Methane Isotopologues in the Subsurface

Biosphere. *Earth Planet. Sci. Lett.* **2021**, *566*, 116960. https://doi.org/10.1016/j.epsl.2021.116960.

(11) Wang, X.; Chen, B.; Chen, L.; Dong, G.; Csernica, T.; Zhang, N.; Liu, J.; Shuai, Y.; Liu, C.-Q.; Xu, Z.; Li, S.-L.; Xu, S. Biogenic Methane Clumped Isotope Signatures: Insights from Microbially Enhanced Coal Bed Methane. *Fuel* **2024**, *365*, 131307. https://doi.org/10.1016/j.fuel.2024.131307.

(12) Haghnegahdar, M. Theoretical Study of Tellurium Isotope Fractionations in Ore-Forming Systems, and Studies of Doubly Substituted Isotopologues of Methane. *PhD Thesis* **2018**, *University of California, Los Angeles (UCLA)*.

(13) York, D.; Evensen, N. M.; Martínez, M. L.; De Basabe Delgado, J. Unified Equations for the Slope, Intercept, and Standard Errors of the Best Straight Line. *Am. J. Phys.* **2004**, *72* (3), 367–375.

(14) Kopec, B. G.; Feng, X.; Posmentier, E. S.; Sonder, L. J. Seasonal Deuterium Excess Variations of Precipitation at Summit, Greenland, and Their Climatological Significance. *J. Geophys. Res. Atmospheres* **2019**, *124* (1), 72–91. https://doi.org/10.1029/2018JD028750.

(15) Manaj, S.; Kim, S.-T. Techniques for Measuring Carbon and Oxygen Isotope Compositions of Atmospheric CO2 via Isotope Ratio Mass Spectrometry. *Rapid Commun. Mass Spectrom.* **2021**, *35* (4), e8995. https://doi.org/10.1002/rcm.8995.

(16) Lloyd, M. K.; Eldridge, D. L.; Stolper, D. A. Clumped 13CH2D and 12CHD2 Compositions of Methyl Groups from Wood and Synthetic Monomers: Methods, Experimental and Theoretical Calibrations, and Initial Results. *Geochim. Cosmochim. Acta* **2021**, *297*, 233–275. https://doi.org/10.1016/j.gca.2020.10.008.

(17) Mueller, E. P.; Sessions, A. L.; Sauer, P. E.; Weiss, G. M.; Eiler, J. M. Simultaneous, High-Precision Measurements of δ^{2}H and δ^{13}C in Nanomole Quantities of Acetate Using Electrospray Ionization-Quadrupole-Orbitrap Mass Spectrometry. *Anal. Chem.* **2022**, *94* (2), 1092–1100. https://doi.org/10.1021/acs.analchem.1c04141.

(18) Gruen, D. S.; Wang, D. T.; Könneke, M.; Topçuoğlu, B. D.; Stewart, L. C.; Goldhammer, T.; Holden, J. F.; Hinrichs, K.-U.; Ono, S. Experimental Investigation on the Controls of Clumped Isotopologue and Hydrogen Isotope Ratios in Microbial Methane. *Geochim. Cosmochim. Acta* **2018**, *237*, 339–356. https://doi.org/10.1016/j.gca.2018.06.029.

(19) Alberty, R. A. Effect of Temperature on the Standard Transformed Thermodynamic Properties of Biochemical Reactions with Emphasis on the Maxwell Equations. *J. Phys. Chem. B* **2003**, *107* (15), 3631–3635. https://doi.org/10.1021/jp022432x.

(20) Sander, R. Compilation of Henry's Law Constants (Version 5.0.0) for Water as Solvent. *Atmospheric Chem. Phys.* **2023**, *23* (19), 10901–12440. https://doi.org/10.5194/acp-23-10901-2023.

(21) Young, E. D.; Rumble III, D.; Freedman, P.; Mills, M. A Large-Radius High-Mass-Resolution Multiple-Collector Isotope Ratio Mass Spectrometer for Analysis of Rare Isotopologues of O2, N2, CH4 and Other Gases. *Int. J. Mass Spectrom.* **2016**, *401*, 1–10.

(22) Young, E.; Orcutt, B.; Daniel, I.; Dasgupta, R. A Two-Dimensional Perspective on CH4 Isotope Clumping. *Deep Carbon* **2019**, *1029*, 388–414.
